# Epithelium dynamics differ in time and space when exposed to the permeation enhancers penetramax and EGTA. A head-to-head mechanistic comparison

**DOI:** 10.3389/fddev.2023.1221628

**Published:** 2023-08-24

**Authors:** D. A. Panou, S. F. Pedersen, M. Kristensen, H. M. Nielsen

**Affiliations:** ^1^ Center for Biopharmaceuticals and Biobarriers in Drug Delivery (BioDelivery), Department of Pharmacy, Faculty of Health and Medical Sciences, University of Copenhagen, Copenhagen, Denmark; ^2^ Section for Cell Biology and Physiology, Department of Biology, Faculty of Science, University of Copenhagen, Copenhagen, Denmark; ^3^ CNS Drug Delivery and Barrier Modelling, Department of Pharmacy, Faculty of Health and Medical Sciences, University of Copenhagen, Copenhagen, Denmark

**Keywords:** penetramax, ethylene glycol tetraacetic acid (EGTA), cell-penetrating peptide, tight junction, cytoskeleton, re-epithelization, permeation enhancer, oral drug delivery

## Abstract

Absorption of therapeutic peptides like glucagon-like peptide or insulin for diabetes therapy upon oral administration is highly restricted by the tight junction (TJ) proteins interconnecting the cells comprising the intestinal epithelium. An approach to improve transepithelial permeation of such biopharmaceuticals via the paracellular pathway is to use functional excipients, which transiently modulate the TJs. Here, we investigated the membrane-interacting peptide, penetramax, and the divalent cation chelator, ethylene glycol tetraacetic acid (EGTA) at different concentrations, to reveal and compare their cellular modes of action when increasing the transepithelial permeation of drug macromolecules. The epithelial integrity was studied in real time along with dextran permeation across differentiated epithelial Caco-2 cell monolayers. TJ protein expression and cytoskeleton organization were investigated during and after exposure to penetramax or EGTA. Based on orthogonal methods, we show that penetramax acts by a mechanism that immediately and transiently widens the paracellular space, resulting in size selective permeant passage and with subsequent reconstitution of the epithelium. At the same time, the expression and organization of different TJ proteins are modulated reversibly. In contrast, the effect of EGTA on modulating the paracellular space is slower and TJ protein unspecific, and without clear permeant size selectivity. Overall, these data provide in-depth insights for understanding intestinal barrier dynamics of importance when evaluating new or existing excipients for oral delivery of biopharmaceuticals, such as peptide therapeutics.

## 1 Introduction

The number of regulatory approvals for biopharmaceuticals are rising (Lau and Dunn, 2018; Walsh, 2018), driven by the increased discovery and application of novel peptide therapeutics for a variety of drug targets. This increases the need to develop competitive and patient-friendly dosage forms complementary to the current injectables (Brayden, 2020). Oral dosing is highly accepted among patients and while several formulations comprising permeation enhancers (PEs) as excipients have entered clinical trials to improve the oral bioavailability of peptide drugs, only two have been approved. These approved formulations utilize medium chain fatty acid (MCFA)-based PEs. Specifically, in Rybelsus^®^ sodium salcaprozate is dosed together with the glucagon-like peptide-1 analog semaglutide for diabetes treatment, and in Mycapssa^®^ sodium caprylate is co-administered with octreotide for acromegaly therapy (Buckley et al., 2018). Despite significant efforts, the achieved bioavailabilities are still low, partly attributed to the challenging environment in the gastrointestinal tract (Artursson and Lundquist, 2020; Brayden, 2020). E.g., while the intestinal epithelium regulates the absorption of nutrients and solutes, it represents a significant biological barrier to absorption of larger molecules such as therapeutic peptides (Brown et al., 2020; Drucker, 2020). Hence, it is crucial to address critical parameters when evaluating the effect of PEs on the intestinal epithelial barrier *in vitro*, facilitating their potential translation to clinical studies, as well as exploring additional PEs beyond MCFAs.

The intestinal epithelium consists of a monolayer of epithelial cells interconnected by cell-cell junctions, with tight junction (TJ) proteins being the primary determinants for permeation through the paracellular pathway (Weber, 2012; Apostolou et al., 2021). TJs are highly organized protein networks of transmembrane and cytoplasmic protein families (Anderson and Van Itallie, 2009; Shen, 2012; Van Itallie and Anderson, 2014). The former includes claudins, TJ-associated MARVEL proteins (e.g., occludin and tricellulin (tric)), and junctional adhesion molecules. Complementing these is, amongst others, the cytosolic scaffold protein family zonula occludens (ZO) that crosslinks most of the transmembrane TJ proteins to the underlying actin cytoskeleton (Monaco et al., 2021). This interconnectedness regulates the movement of ions and solutes through the paracellular space (González-Mariscal et al., 2000; Van Itallie and Anderson, 2006; Van Itallie et al., 2009). Thus, an approach to improve transepithelial peptide drug permeation and hence the bioavailability of orally administered peptide drugs is to transiently modulate the TJ and cytoskeleton dynamics using PEs (Krug et al., 2013; Bocsik et al., 2016; Krug et al., 2017; Neuhaus et al., 2018). PEs can act transcellularly and/or paracellularly. Irrespective of the exact mechanism, a PE should be sufficiently efficient and safe to use implying that the effect should be rapid, selective, and reversible.

Examples of paracellularly acting PEs comprise the MCFA sodium caprate that interacts with the cell membrane leading to modulation of TJs and the paracellular space (Krug et al., 2013) and the small molecule and chelating agent, ethylene glycol tetraacetic acid (EGTA) known for its effect on epithelial barrier properties due to Ca^2+^ depletion (Raiman et al., 2003). EGTA is an analog of ethylenediaminetetraacetic acid (EDTA), which is included as one of more components in the POD™ technology that have been tested in patients for oral insulin delivery (Study Record, 2023), although it recently failed to meet clinical endpoints in a phase 3 study (Study Record, 2023). As EGTA is significantly more selective towards Ca^2+^ ions over Mg^2+^ ions, as compared to EDTA (Caldwell and Cuthbert, 1970), we selected EGTA as comparator in the present study. Previous studies found EDTA to effectively enhance permeation when applied in the one-digit mM range (Noach et al., 1993).

Here, we investigate the effects of penetramax, a membrane-interacting peptide-based enhancer, and analog of penetratin, a cell-penetrating peptide, which we and others previously reported to enhance peptide delivery *in vitro* and *in vivo* (Kamei et al., 2013a; Diedrichsen et al., 2021). *In vitro*, effective concentrations of penetramax are in the two-digit µM range (Diedrichsen et al., 2023). We compare the findings head-to-head to those observed with EGTA with the aim to analyze the underlying cellular mechanisms that regulate TJ and cytoskeleton dynamics for both enhancers. Thus, we aimed to address critical parameters for evaluating PE effects on epithelial barrier dynamics, permeant size selectivity, and epithelial barrier recovery.

## 2 Materials and methods

### 2.1 Materials

Penetramax (KWFKIQMQIRRWKNKR, 2,247 Da) was synthesized by Synpeptide (Shanghai, China) with a purity >95%. EGTA ([-CH_2_OCH_2_CH_2_N(CH_2_CO_2_H)_2_]_2_) with a purity >97%, bovine serum albumin (BSA) with a purity >98%, fluorescein isothiocyanate (FITC)-labeled dextrans 4 and 10 (FD4 and FD10, average Mw: 4,000 g/mol and 10,000 g/mol), phenazine methosulfate (PMS), polysorbate 20, Triton^®^ X-100, EDTA, Trizma^®^, sucrose, Tergitol™ (NP-40) and skim milk powder for microbiology were obtained from Sigma-Aldrich (Merck KGaA, Darmstadt, Germany). 4-(2-hydroxyethyl)-1-piperazine-1- ethanesulfonic acid (HEPES) was purchased from PanReac AppliChem (Darmstadt, Germany). Hank’s balanced salt solution containing 1 mM Ca^2+^ and 0.9 mM Mg^2+^ (HBSS), Dulbecco’s phosphate-buffered saline (PBS, pH 7.2), Dulbecco’s modified Eagle’s medium (DMEM), penicillin/streptomycin, L-glutamine, non-essential amino acid solution (NEAA), and trypsin-EDTA, were purchased from Merck (Kenilworth, NJ, United States). ECL^®^ immunoblotting detection reagents were obtained from GE Healthcare (Cardiff, United Kingdom). Phosphate-buffered saline, Tris-buffered saline (TBS), resin kit medium, 1,2 propylene oxide, and glutaraldehyde 25% (v/v) were from VWR (Radnor, PA, United States). 2-(N-morpholino) ethanesulfonic acid (MES) running buffer, 3-morpholinopropane-1-sulfonic acid sodium dodecyl sulfate (SDS) running buffer (MOPS), 16% (w/v) paraformaldehyde (PFA) aqueous solution methanol free (Alfa Aesar), 4,6-diamidino-2-phenylindole (DAPI), Alexa Fluor^®^ 555 phalloidin, Pierce™ BCA protein kit, and Mount Medium Scientific™ Sadon™ Immu-Mount™ were purchased from Thermo Fisher Scientific (Waltham, MA, United States). Likewise, the antibodies rabbit anti-zonula occludens-1 (ZO-1) (#61-7300, RRID:AB_2533938), rabbit anti-occludin (#71-1500; RRID:AB_2533977), rabbit anti-tricellulin (tric) (#700191; RRID:AB_2532298), rabbit anti-claudin-1 (#717800; RRID:AB_2533997), rabbit anti-claudin-7 (#349100; RRID:AB_2533190), mouse anti-claudin-2 (#325600; RRID:AB_2533085), mouse anti-claudin-4 (#329400; RRID:AB_2533096), goat anti-mouse IgG (#31430; RRID:AB_228307), goat anti-rabbit (#31460; RRID:AB_22834), goat anti-rabbit IgG (H + L) Trial Superclonal™ secondary antibody Alexa Fluor 488 (#A27034; RRID:AB_2536097) and goat anti-mouse IgG (H + L) highly cross-adsorbed secondary antibody Alexa Fluor Plus 488 (#A32723; RRID:AB_2633275) were from Thermo Fisher Scientific. Rabbit anti-β actin antibody (#ab8227; RRID:AB_2305186) was purchased from Abcam (Burlingame, CA, United States). Abberior^®^ STER RED NHS (#43354) and Abberior MOUNT^®^ media were purchased by Abberior (Göttingen, Germany). Fetal bovine serum (FBS) was purchased from PAA laboratories (Brøndby, Denmark). 3-(4,5-dimethylthiazol-2-yl)-5-(3-carboxymethoxyphenyl)-2-(4-sulfophenyl)-2H-tetrazolium) (MTS) was obtained from Promega (Madison, WI, United States). Osmium tetroxide 4% (w/v) solution was from Polysciences Europe (Hirschberg an der Bergstrasse, Germany) and potassium ferricyanide from Fluka^®^ Analytical (Buchs, Switzerland). Ultrapure water was obtained from a PURELAB flex 4 system (ELGA LabWater, High Wycombe, United Kingdom).

### 2.2 Cell culture

The human colorectal adenocarcinoma cell line Caco-2 (American Type Culture Collection (ATCC) #HTB-37^;^ RRID:CVCL_0025, Manassas, VA, United States) was maintained in T175 cm^2^ flasks (Merck) in high-glucose DMEM supplemented with 10% (v/v) FBS, 0.1 mM NEAA, 2 mM L-glutamine, 90 IU/mL penicillin, and 90 μg/mL streptomycin, in a humidified atmosphere (5% CO_2_, 95% O_2_, 37°C). The cell culture medium was changed every second day. The cells were passaged using trypsin-EDTA at 80%-85% confluency once a week. For experimental use, the Caco-2 cells were seeded (1 × 10^5^ cells/insert) on polycarbonate Corning Transwell^®^ filter inserts (model 3140, 0.4 µm pore size, 1.12 cm^2^, Corning, NY, United States) and cultured in a humidified atmosphere (5% CO_2_, 95% O_2_, 37°C) for 21 days in cell culture medium with medium change every second day and the day before an experiment. Passage 3–11 were used for experiments. The initial transepithelial electrical resistance (TEER) values for Caco-2 cells grown on filter inserts included in the studies ranged from 226 to 400 Ω × cm^2^ with similar TEER values within each passage and the main variation observed between passages.

### 2.3 Test samples

Stock solutions (1 mM) of penetramax were prepared in ultrapure water based on weighed powder including the presence of trifluoroacetic acid as counterion. The desired test concentrations of 25, 55, 75, and 95 µM penetramax were obtained upon appropriate dilution in 10 mM HEPES-HBSS (Ca^2+^/Mg^2+^) pH 7.4 (referred to as hHBSS). Stock solutions of EGTA (100 mM, pH 7.8) were prepared in ultrapure water and further diluted in hHBSS prior use to achieve the desired final concentrations of 1, 3, 5, and 7 mM EGTA.

### 2.4 Transepithelial electrical resistance (TEER)

#### 2.4.1 End-point TEER measurements

The Caco-2 cell monolayers were washed twice with 37°C hHBSS or hHBSS supplemented with 0.05% (w/v) BSA on both the apical and basolateral sides, respectively. The monolayers were equilibrated to room temperature (RT) for 20 min in the last washing volume prior to TEER measurements using an ENDOHM 12-G cup connected to an EVOM volt ohmmeter (World Precision Instruments, Sarasota, FL, United States). PE concentrations in the ranges 1–10 mM for EGTA and 10–100 µM for penetramax (data not shown) were screened for their effect on TEER after 3 h incubation as described below, and four concentrations were selected for each of the PEs. The buffer was replaced with 0.35 mL of test sample consisting of 25, 55, 75, or 95 µM penetramax or 1, 3, 5, or 7 mM EGTA in hHBSS in the apical compartment and with 1 mL hHBSS supplemented with 0.05% (w/v) BSA in the basolateral compartment. The monolayers were transferred to a 37°C shaking table with orbital shaking (50 rpm) (Thermo MaxQ 2000, Thermo Fischer Scientific, West Palm Beach, FL, United States with custom-made temperature isolation). After 3 h of exposure, the cells were left at RT to equilibrate for 20 min prior to TEER measurements. After TEER measurements, the Caco-2 cell monolayers were washed twice with 37°C hHBSS or hHBSS supplemented with 0.05% (w/v) BSA on the apical and basolateral sides, respectively, and kept in cell culture medium for recovery for additional 18 h in an incubator (5% CO_2_, 95% O_2_, 37°C). Subsequently, and after 20 min equilibration to RT, the monolayers were subjected to TEER measurements. The epithelial integrity was calculated according to Eq. 1.

$$\frac{\frac{\left(TEER\;test\;sample,\;after\right)}{\left(TEER\;test\;sample,\;before\right)}\times 100\%}{\frac{\left(TEER\;buffer,\;after\right)}{\left(TEER\;buffer,\;before\right)}\times 100\%}\times 100\%$$ (1)

#### 2.4.2 Real time TEER measurements

To test the effect of 25, 55, 75, and 95 µM penetramax and 1, 3, 5, and 7 mM EGTA on the integrity of Caco-2 cell monolayers in real time, a CellZscope (nanoAnalytics, Münster, Germany) was used to record TEER every 20 min. 20 days post-seeding, Caco-2 cells grown on filter supports were transferred to CellZscope chambers with 1 mL and 1.5 mL cell culture medium in the apical and basolateral compartments, respectively. The Caco-2 cell monolayers were placed in an incubator (37°C and 5% CO_2_ humidified air) and left to equilibrate overnight (ON). Then, the cell monolayers were washed twice with hHBSS (1 mL) and hHBSS supplemented with 0.05% (w/v) BSA (1.5 mL) on the apical and basal sides, respectively, and left to equilibrate for 1 h (37°C and 5% CO_2_ humidified air). When testing the effect of different penetramax and EGTA concentrations, 20–45 µL of the excipient stock was spiked into the apical hHBSS buffer (maintaining a total volume of 800 µL in the apical compartment) followed by 3 h exposure with orbital shaking (50 rpm) at 37°C. The test samples were gently removed, and the cell monolayers were washed twice with hHBSS and hHBSS supplemented with 0.05% (w/v) BSA on the apical and basolateral sides, respectively, and placed in cell culture medium for subsequent TEER measurements. Effects are presented as percent of TEER upon equilibration at 37°C normalized to that of cell monolayers in buffer with t_0_ being the time of excipient addition and recovery initiation (Eq. 2). TEER values are always measured at 37°C and compared at every recorded cycle occurring every 20 min. Eq. 2 thus describes a continuous measurement of TEER changes over time (during exposure and recovery) compared to the baseline after equilibration.

$$\frac{\frac{\left(TEER\;test\;sample\;during\;exposure\right)}{\left(TEER\;test\;sample\;at\;t_{0}\right)}\times 100\%}{\frac{\left(TEER\;buffer\;during\;exposure\right)}{\left(TEER\;buffer\;at\;t_{0}\right)}\times 100\%}\times 100\%$$ (2)

### 2.5 Metabolic activity

The metabolic activity of the 21-day post-seeding differentiated Caco-2 cell monolayers was evaluated after 3 h incubation with buffer, with 25, 55, 75, or 95 µM penetramax, or with 1, 3, 5, or 7 mM EGTA as well as upon 21 h recovery using the MTS-PMS assay. Following penetramax or EGTA exposure, the monolayers were washed twice with 37°C hHBSS or hHBSS supplemented with 0.05% (w/v) BSA on the apical or basolateral side, respectively. The buffer was replaced with 0.35 mL of MTS-PMS (240 μg/mL MTS and 2.4 μg/mL PMS) in hHBSS in the apical compartment and 1 mL hHBSS supplemented with 0.5% (w/v) BSA in the basolateral compartment. The inserts were transferred to a 37°C shaking table (Thermo Fisher Scientific with temperature isolation) applying orbital shaking (50 rpm) for 60-90 min 2 × 100 µL samples from the apical compartment were transferred to a clear-bottom 96-well plate (Greiner, Pleidelsheim, Germany), and the absorbance was measured at 490 nm using a FLUOstar OPTIMA plate reader (BMG Labtech, Ortenberg, Germany). The relative metabolic activity was calculated using Eq. 3:

$$Relative\;metabolic\;activity=\frac{\left(A-C\right)}{\left(B-C\right)}\times 100\%$$ (3)

where A is the absorbance of samples taken from the apical side of Caco-2 cell monolayers exposed to penetramax or EGTA, B is the absorbance of samples taken from the apical side of Caco-2 cell monolayers in hHBSS, and C is the absorbance of MTS-PMS reagent included as background control, instead of cells exposed to e.g., 0.2% (w/v) sodium dodecyl sulfate as a positive control since SDS lyses and/or fully detaches the cells from the filter insert leaving only buffer with the MTS-PMS reagent as the C sample.

### 2.6 FITC-labeled dextran (FD) permeation

The Caco-2 cell monolayers were washed and the TEER measured as described for the end-point TEER measurements. The permeation study was done in the same wells as used for the TEER measurement. Then, the buffer was replaced with 0.37 mL of 25, 55, 75, or 95 µM penetramax or 1, 3, 5, or 7 mM EGTA together with 1 mg/mL FD4 or FD10 kDa in hHBSS in the apical compartment. 1 mL hHBSS supplemented with 0.05% (w/v) BSA was used in the basolateral compartment. The inserts were transferred to a 37°C shaking table (Thermo Fischer Scientific, with temperature isolation) employing orbital shaking (50 rpm) for 3 h. 20 μL donor samples were withdrawn from the apical compartment at time points 0 and 180 min and 100 μL samples were withdrawn from the basolateral compartment at time points 30, 60, 90, 120, 150, and 180 min and kept protected from light in a black clear-bottom 96-well plate (Nunc, Roskilde, Denmark) until analysis immediately after termination of the experiment. The 100 µL sampling volumes were replaced with hHBSS supplemented with 0.05% (w/v) BSA. FD permeation was evaluated using a SPECTROstar fluorescence plate reader (BMG LABTECH, Offenberg, Germany) with excitation/emission set to 480/520 nm.

The apparent permeability coefficient, P_app_, was calculated according to Eq. 4:

$$P_{app}=\frac{dQ/\;dt}{\left(A\times C_{0}\right)}$$ (4)

where P_app_ (cm/s) is the apparent permeability, dQ/dt (µg/s) is the steady-state flux, A is the area of the filter insert (1.12 cm^2^), and C_0_ (µg/mL) is the initial donor sample concentration. The P_app_ presented was calculated for t = 30-90 min.

### 2.7 Tight junction and cytoskeleton visualization using confocal laser scanning microscopy (CLSM) and stimulation emission depletion (STED) microscopy

Changes in TJ localization and morphology of Caco-2 cell monolayers were analyzed after 3 h incubation with 25, 55, 75, or 95 µM penetramax or 1, 3, 5, or 7 mM EGTA, and after 21 h subsequent recovery in growth medium. The Caco-2 cell monolayers were washed twice with hHBSS and hHBSS supplemented with 0.05% (w/v) BSA apically and basolaterally, respectively. The cell monolayers were fixed with 3.7% (v/v) paraformaldehyde in PBS for 15 min at RT. After two washing steps with PBS, the monolayers were permeabilized with 0.2% (v/v) Triton™ X-100 in PBS for 15 min. Next, they were blocked with 4% (w/v) BSA in PBS (blocking buffer) for 40 min and separated from the plastic inserts before incubation with the primary antibodies (directed against claudin-1 (1:200), −2 (1:180), −4 (1:180), −7 (1:180), ZO-1 (1:150), occludin (1:200), and tric (1:180)) and phalloidin (1:100), to stain the cytoskeleton filamentous actin (F-actin), in blocking buffer at 4°C ON with horizontal shaking (100 rpm). The cell monolayers were washed once with PBS (RT) and incubated for 45 min in blocking buffer at RT, and then incubated with secondary antibody (1:200) diluted in blocking buffer for 1 h at RT with horizontal shaking (100 rpm). The nuclei were stained with DAPI (1:10000) for 15 min. Monolayers then were washed twice with PBS before being mounted on microscope slides using Immuno Mount^®^ for CLSM and Aberrior Mount^®^ for STED microscopy using 1.5H thickness coverslips. Images were captured using a Zeiss LSM710 confocal microscope (Carl Zeiss, Jena, Germany) equipped with a 405 nm diode laser for DAPI, 488 nm argon laser for Alexa Fluor^®^ 488, and a 561 nm diode-pumped solid-state laser for phalloidin using a PMT detector for capturing the DAPI signal and a GaAsP detector for the Alexa Fluor^®^ 488 and Alexa Fluor^®^ 555 signals, using Nyquist sampling. For STED imaging, a STEDYCON (Abberior Instruments, Göttingen, Germany) system confocal microscope (Carl Zeiss, Jena, Germany) equipped with 561 nm and 640 nm pulsed excitation lasers and a 775 nm pulsed depletion laser using 20 nm pixels. For image acquisition, a Plan-Apochromat 63×/1.4 N.A. oil immersion objective (confocal microscopy) and a ×100/1.46 N.A. oil immersion objective (STED microscopy) was used for scanning in the lateral and axial directions. When necessary, the intensity of the images was adjusted for brightness and contrast and the images were displayed as maximum projections using the ImageJ^®^ software program (Schneider et al., 2012). To prepare the microscopy figures, the open-source plug-in ScientiFig for ImageJ^®^ was used (Aigouy and Mirouse, 2013). Deconvolution of STED images was performed with Huygens software.

### 2.8 Ultrastructural analysis using transmission electron microscopy (TEM)

The ultrastructure of the Caco-2 cell monolayers was analyzed after 3 h incubation with 25, 55, 75, or 95 µM penetramax or 7 mM EGTA, and after 21 h subsequent recovery in growth medium, using TEM. The monolayers were washed twice with PBS and subsequently fixated with 2% (v/v) glutaraldehyde in 0.05 M in PBS at RT (for at least 24 h), prior to washing twice with 0.12 M cacodylate for 20 min. The cells were subjected to post-fixation in 1% (w/v) osmium tetroxide, 0.05 M potassium ferricyanide, and 0.12 M cacodylate for 1 h at RT and dehydrated stepwise in a graded ethanol series (70%, 96%, and absolute ethanol) thrice for 15 min for each step. The monolayers were infiltrated in propylene oxide and subsequently in 1/3, 1/1, and 3/1 proportions of Epon (TAAB, T031)/polypropylene oxide gradients for 40 min per gradient and finally in pure Epon resin for 2 h under gentle rotation. Resin polymerization was performed ON at 60°C. Ultra-thin sections, approximately 60 nm thick, were cut with an Ultracut 7 microtome (Leica, Vienna, Austria) and collected on copper grids with Formvar supporting membranes, stained with uranyl acetate and lead citrate, and subsequently examined using a Philips CM 100 Transmission EM (Philips, Eindhoven, Netherlands), operated at an accelerating voltage of 80 kV. Digital images were recorded with an OSIS Veleta digital slow scan 2k × 2k CCD camera and the ITEM software package.

### 2.9 Protein expression analysis using immunoblotting

Changes in TJ protein expression was evaluated after 3 h exposure to 25, 55, 75, or 95 µM penetramax or 1, 3, 5, or 7 mM EGTA, and after 21 h recovery in growth medium, using immunoblotting. The Caco-2 cell monolayers were washed twice with ice-cold hHBSS, and the cells were lysed with 2% (v/v) NP-40 cell lysis buffer (10 mM Tris-HCl, pH 7.4, 0.25 M sucrose, 1 mM EGTA, 1 mM EDTA, 2% (v/v) NP-40) supplemented with protease inhibitor cocktail (Complete Mini, Roche Diagnostics, Basel, Switzerland) for 30 min on ice. The cell lysates were subjected to centrifugation for 15 min at 1500 g and 0°C (Eppendorf^®^ microcentrifuge, Eppendorf Nordic, Hørsholm, Denmark). The supernatants were stored at −20°C or −80°C until use. The total protein in the lysates was quantified using the BCA protein quantification kit (Pierce™, Thermo Scientific) as directed by the manufacturer. 12.5-20 µg protein were loaded onto NuPAGE™ 4%-12% Bis-Tris gels (Novex Life Technologies, Invitrogen, Thermo Scientific) for separation using a miniXCell^®^ device (Novex Life Technologies). The protein bands were electro-transferred onto a polyvinylidene difluoride membrane using Trans-Blot^®^ Turbo™ Mini PVDF Transfer Packs (Bio-Rad, Hercules, CA, United States) and the Trans-Blot Turbo Transfer Starter System (Bio-Rad). The membrane was blocked with 5% (v/v) skim milk in TBS with 0.1% (v/v) polysorbate 20 for 1 h before incubation with primary antibodies [anti-claudin-1, anti-claudin-2, anti-claudin-4, anti-claudin-7, anti-tric (all 1:1000), anti-ZO-1 and anti-occludin (1:1500)] at 4°C ON. The membrane was washed thrice with TBS with 0.1% (v/v) polysorbate 20 prior incubation with horseradish peroxidase-conjugated secondary antibody (1:4000 for monoclonal anti-mouse and 1:7000 for polyclonal anti-rabbit apart from occludin, for which the dilution factor was 1:8500) for 1 h and washed five times with 0.1% (v/v) polysorbate 20 in TBS. The membranes were visualized using an ECL^®^ Prime Western blotting System in a Fluorochem Q imaging system (Alpha Innotech, Santa Clara, CA, United States). Equal protein loading was assessed by membrane re-probing with anti-β actin (1:5000) antibody as described above. The only difference was that the incubation for the primary antibody was 1 h, and the dilution of the secondary antibody was 1:11000. The densitometry analysis was performed with Fluorochem Q software for semi-quantifying the bands corresponding to the TJ protein under investigation and normalized against bands for anti-β actin as a loading control.

### 2.10 Data and statistical analysis

Data analysis was performed with Microsoft Excel 2010 software (RRID:SCR_016137; Microsoft, Houston, TX, United States) and GraphPad Prism 7 (RRID:SCR_002798; GraphPad Software, San Diego, CA, United States). Data are presented as mean with standard error of mean (SEM) apart from TJ protein expression from immunoblots for which standard deviation (SD) were used. “n” represents the number of technical replicates within each passage of cells and “N” represents the number of independent biological experiments. Statistical analysis was performed by GraphPad Prism 7 using one-way analysis of variance (ANOVA) followed by Dunnett’s T3 multiple comparison for data with unequal SDs.

## 3 Results

### 3.1 Penetramax and EGTA decrease epithelial integrity without corresponding effects on cell metabolic activity

**FIGURE 1:**
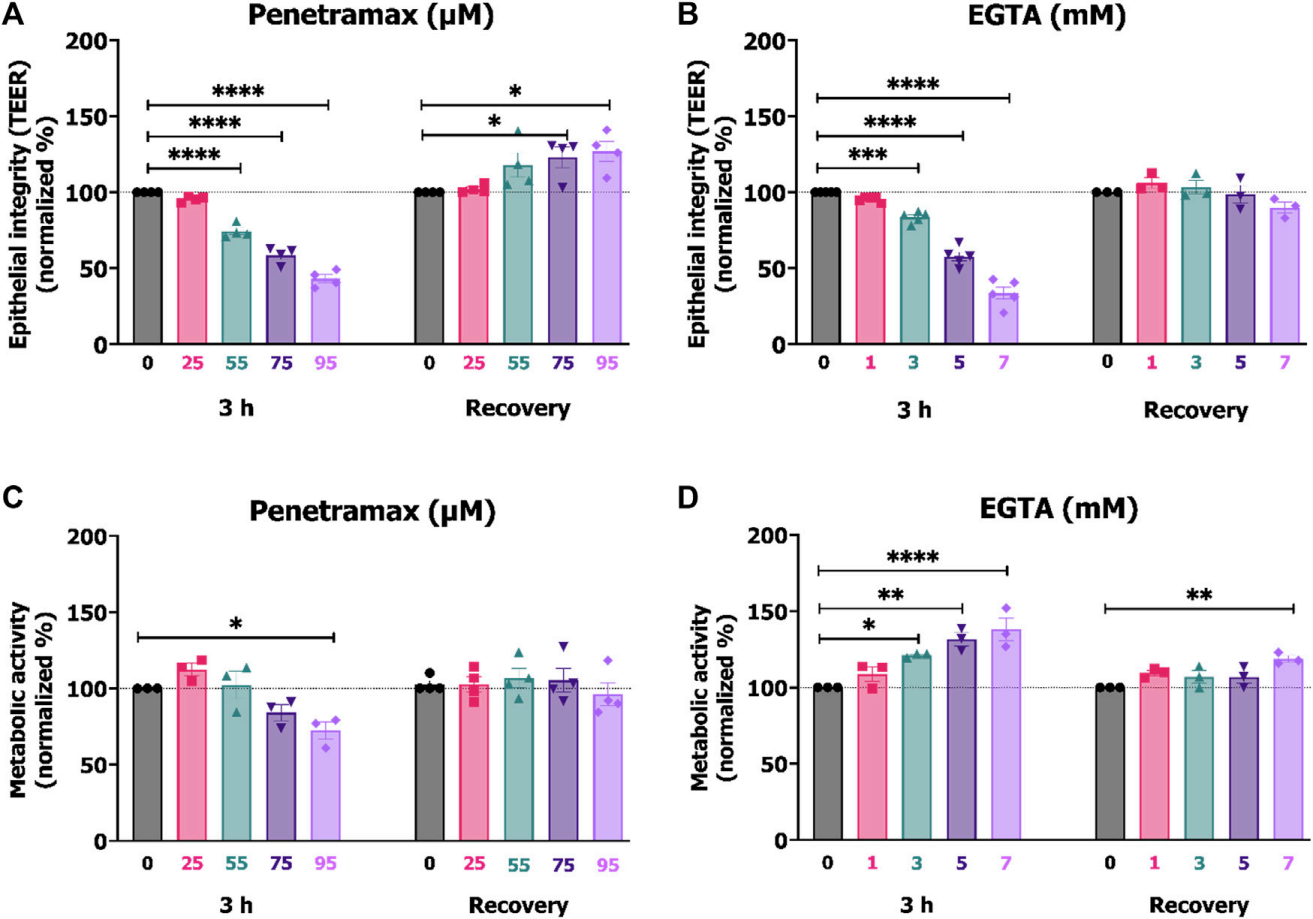
Comparable TEER reduction between 25, 55, 75, 95 µM penetramax and 1, 3, 5, 7 mM EGTA after 3 h exposure. Effects on the integrity and metabolic activity were transient upon recovery. End-point TEER measurements of Caco-2 cell monolayers exposed to **(A)** penetramax and **(B)** EGTA for 3 h and subsequent recovery in cell culture medium for 21 h. N = 3–4, *n* = 3–6. Metabolic activity in Caco-2 cell monolayers exposed to **(C)** penetramax and **(D)** EGTA for 3 h and after subsequent recovery in cell culture medium for 21 h of exposure. N = 3–5, *n* = 2–6 **(A–D)** Horizontal lines represent baseline values. Results are shown as mean ± SEM in comparison to buffer. *) *p* < 0.05, **) *p* < 0.01, ***) *p* < 0.001, ****) *p* < 0.0001.

We used the TEER of the differentiated Caco-2 cell monolayers to provide insight into the effects of penetramax and EGTA on the epithelial barrier integrity. A screening of various concentrations was performed, and subsequent investigations focused on excipient concentrations that after 3 h exposure resulted in approximately 95%, 80%, 60%, and 40% of the initial TEER. Penetramax decreased the TEER to 96%, 75%, 59%, and 43% upon exposure to 25, 55, 75, and 95 µM penetramax, respectively (Figure 1A). In comparison, the TEER decreased to 96%, 84%, 58%, and 34% upon exposure to 1, 3, 5, and 7 mM EGTA, respectively (Figure 1B). As an indicator of the effect of penetramax and EGTA on cellular viability, the metabolic (dehydrogenase) activity was assessed after 3 h exposure. The metabolic activity was not reduced to the same extent as the TEER by penetramax exposure, and only resulted in a significant decrease by 28% after exposure to the highest penetramax concentration (Figure 1C). In contrast, exposure to EGTA resulted in a concentration-dependent stimulation of the metabolic activity corresponding to increases of 23%, 32%, and 38% for 3, 5, and 7 mM EGTA, respectively, when compared to the buffer exposed cells (Figure 1D).

After 3 h exposure to penetramax or EGTA, the cell monolayers were washed and subjected to 21 h recovery in cell culture medium. Overall, both the integrity and the cellular metabolic activity were reestablished (Figures 1A–D), yet the cell monolayers exposed to 75 and 95 µM penetramax displayed slightly increased TEER values by 23% and 27%, respectively. This was also the case for the metabolic activity of cell monolayers exposed to 7 mM EGTA (19%).

These results indicate that penetramax and EGTA exert similar and reversible effects on epithelial integrity. However, upon recovery for 21 h, the epithelial integrity was at baseline values only for EGTA, while increased for penetramax. The metabolic activity was at baseline for penetramax after 21 h recovery.

### 3.2 Penetramax and EGTA induce contraction of the perijunctional actomyosin ring, but display different morphological effects and degrees of cytoskeletal restoration

**FIGURE 2:**
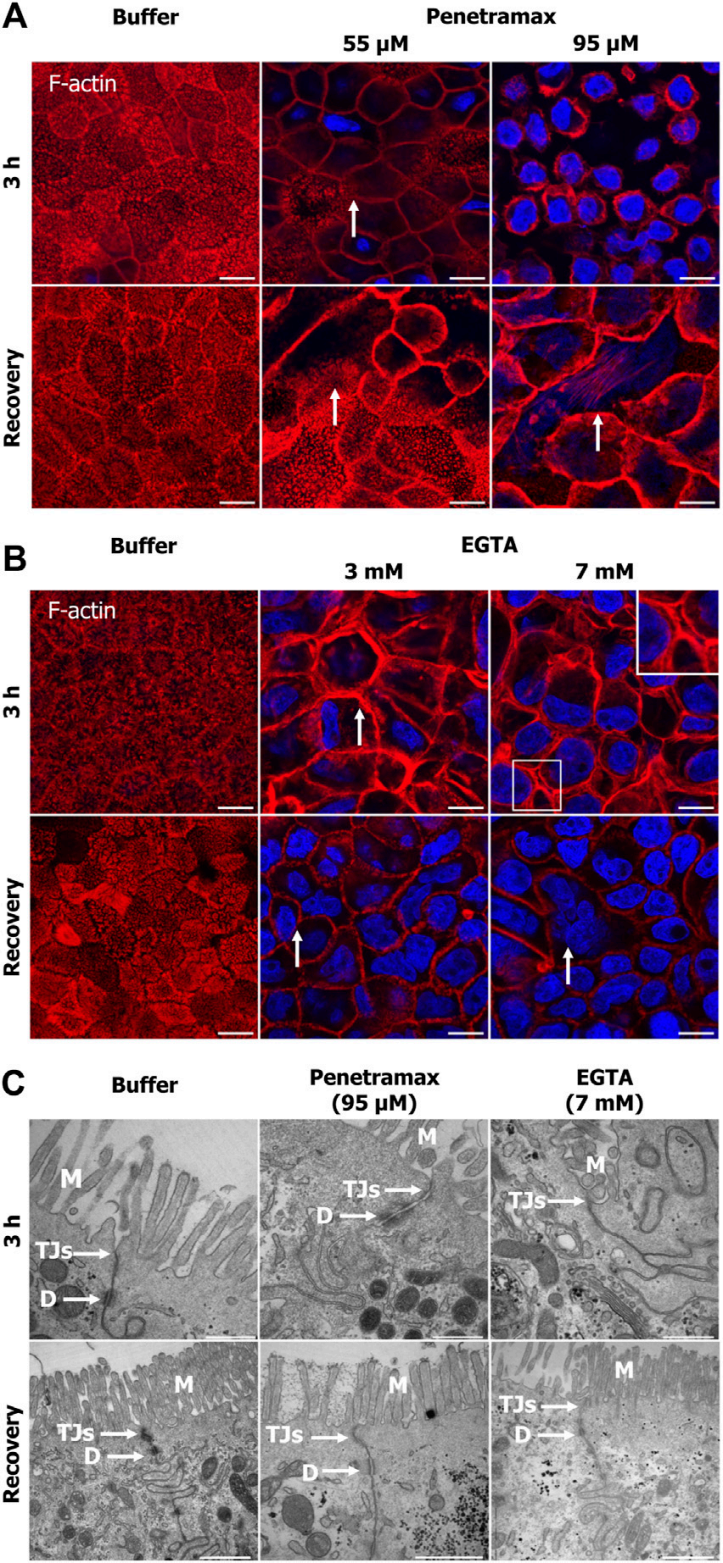
Penetramax and EGTA contract the actin cytoskeleton (F-actin) after 3 h of exposure with paracellular space widening, cytoskeletal reconstitution, and tight junction sealing after subsequent recovery. Representative confocal images of rhodamine-conjugated phalloidin for staining the F-actin. Images presented as maximum intensity projections of Caco-2 cell monolayers exposed to **(A)** penetramax (zoom, selected from Supplementary Figure S1C) and **(B)** EGTA (zoom, selected from Supplementary Figure S1D) for 3 h (top row, 3 planes) and after subsequent recovery in cell culture medium for 21 h (bottom row, three planes). Nuclei stained in blue. N = 2 and *n* = 1, except for penetramax 3 h exposure where N = 3, *n* = 1. Scale bar 10 µm **(C)** Representative transmission electron micrographs of Caco-2 cell epithelium exposed to 95 µM penetramax and 7 mM EGTA for 3 h (top row) and after subsequent recovery in cell culture medium for 21 h (bottom row). M: Microvilli, TJs: Tight junctions, D: Desmosomes. Scale bar 500 nm for 3 h and 1 µm for recovery. N = 2, *n* = 1. Images are zoom-in of selected images in Supplementary Figure S1C **(A, B)** and Supplementary Figure S2 **(C)**.

To investigate cytoskeletal changes underlying the effects induced by penetramax and EGTA on the epithelial integrity and metabolic activity, penetramax and EGTA exposed cell monolayers were stained with phalloidin. This was done immediately after exposure and following recovery to investigate the changes in filamentous (F)-actin. Buffer (Figure 2) and the lowest concentration of penetramax (25 µM) and EGTA (1 mM) exposed cells (Supplementary Figure S1B) revealed the characteristic apical tight perijunctional actomyosin ring. In contrast, exposure to the high concentrations of penetramax (75 and 95 µM) and EGTA (5 and 7 mM) induced F-actin reorganization and contraction (Figures 2A, B; Supplementary Figures S1B–F) as well as a rounded cellular morphology (Supplementary Figures S1B, C). At intermediate concentrations (55 µM penetramax and 3 mM EGTA), the cellular morphology was also altered, however, not to the same extent (Figures 2A, B). After exposure to 55 µM penetramax (Figure 2A), the apical membranes with the characteristic brush border structure of the microvilli appeared stretched, while with 3 mM EGTA (Figure 2B) the brush border appeared disassembled. Despite the cytoskeletal re-organization, cell monolayers showed signs of reconstitution with some phenotypic changes after the recovery period. A smaller cell height-to-area ratio was observed compared to the buffer exposed cells, corresponding to stretched apical membranes and a seemingly reduced cell volume for the intermediate penetramax concentration (Figure 2A; Supplementary Figure S1C), while for the highest penetramax concentration, the formation of stress fibers on the basal side of the Caco-2 cell monolayers was observed (Supplementary Figures S1C, S1E). A similar cytoskeletal reorganization was observed after exposure to EGTA, yet some cells appeared to be devoid of actin filaments (Figure 2B arrows).

Also, Caco-2 cell monolayers were subjected to TEM imaging to examine potential effects of the excipients on the monolayer ultrastructure. Upon exposure to the high concentration of penetramax (95 µM), a widening of the TJs and desmosomes was observed along with the formation of cell multilayers (Figure 2C; Supplementary Figure S2), whereas exposure to the highest concentration of EGTA (7 mM) resulted in a complete opening of the paracellular space and de-localization of TJs from the lateral to the basal compartment (Supplementary Figure S2A bottom row). After recovery from penetramax exposure, the epithelium morphology reverted to a monolayer, though seemingly smaller in cell volume compared to the volume of buffer exposed cell monolayers (Supplementary Figure S2 bottom row). Notably, TJs were present at the apical surface and sealed the paracellular space (Figure 2C, bottom row). Intriguingly, although cytoskeletal reconstitution was observed after exposure to 7 mM EGTA, multilayers were formed (Supplementary Figure S2) and TJs were largely absent.

Collectively, these data show that despite both excipients seemingly contract the actomyosin perijunctional ring, penetramax induced widening of the paracellular space while EGTA fully opened it.

### 3.3 Penetramax and EGTA both affect the dynamics of the cell monolayer integrity, but only penetramax facilitates permeant size selectivity

**FIGURE 3:**
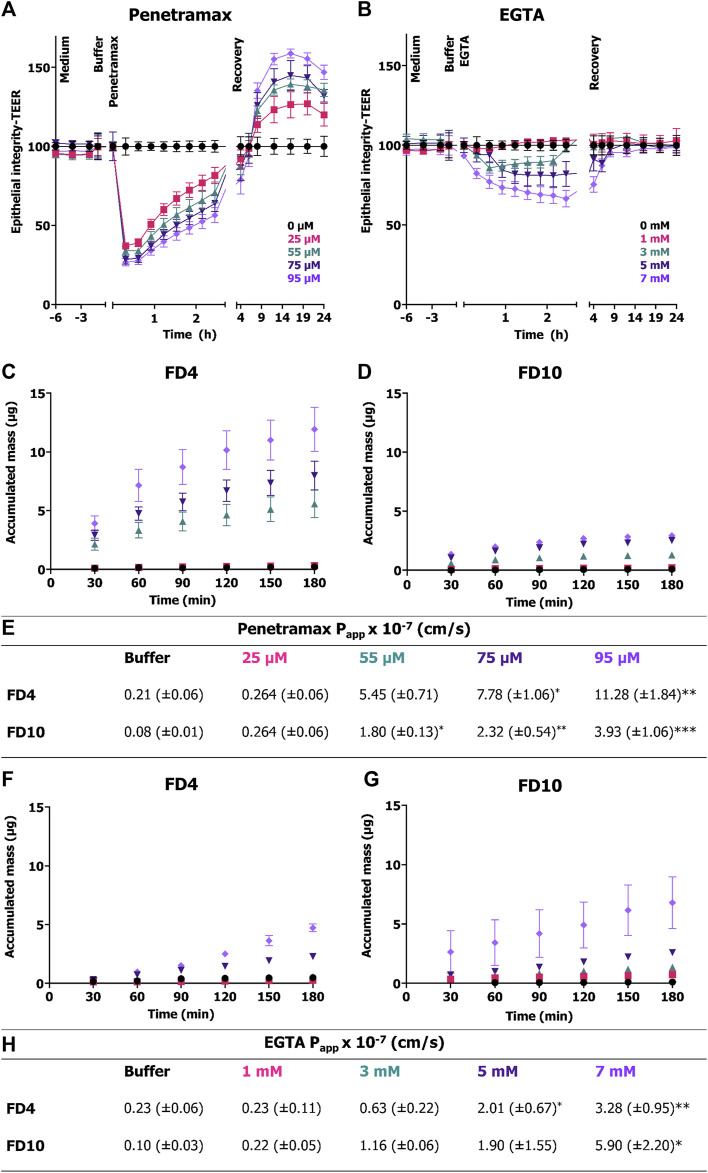
Immediate and transient effect of penetramax during exposure in contrast to the slower and gradual effect of EGTA on epithelial integrity and their corresponding permeability effects. Real time TEER measurements for cell monolayers exposed to **(A)** penetramax and **(B)** EGTA. Permeation of **(C)** FD4 and **(D)** FD10 across Caco-2 cell monolayers exposed to penetramax and **(E)** calculated P_app_ values from steady state flux between 30 and 90 min. N = 2–3, *n* = 3–6. Permeation of **(F)** FD4 and **(G)** FD10 across Caco-2 cell monolayers exposed to EGTA and **(H)** calculated P_app_ values from steady state flux between 30- and 90-min. N = 2-3, *n* = 2-4. All experiments were done in triplicates (N = 3), yet some of the first time point samples in the buffer and low permeation enhancer samples displayed no signal over the limit of detection leading to exclusion of those samples, and thus reduced N and n (for details, see Supplementary Table S1). Results are shown as mean ± SEM. *) *p* < 0.05, **) *p* < 0.01, ***) *p* < 0.001.

Next, epithelial integrity was monitored in real time to understand the cellular mechanisms involved in the observed cytoskeletal reorganization and reconstitution resulting from exposure to penetramax or EGTA. TEER was recorded every 20 min by impedance spectroscopy for 24 h following the exposure to penetramax (Figure 3A) or EGTA (Figure 3B). For penetramax, a fast-acting mechanism was observed, with a significant, immediate, and concentration-dependent, yet transient, decrease in TEER. Reversibility was gradual and continuous during the exposure to penetramax. Consistent with what was observed in the endpoint measurements (Figure 1A), the increase in TEER continued during recovery in a concentration-dependent manner, peaking at values 27%–59% higher than that recorded in the buffer exposed cell monolayers (Figure 3A). In contrast, EGTA exposure resulted in a slower, concentration-dependent decrease in TEER, followed by a plateau phase; except for highest concentration (7 mM), where the TEER continued to decrease to a maximum decrease of 66% after 3 h EGTA exposure. Upon replacing the EGTA samples with cell culture medium, the Caco-2 cell monolayers regained their baseline TEER within 1–2 h (Figure 3B).

To determine the impact of penetramax and EGTA on size-selectivity of epithelial permeability, the permeation of FD4 and FD10 across Caco-2 cell monolayers was assessed (Figures 3C–H). During exposure to penetramax concentrations higher than 25 μM, the permeation of both FD4 and FD10 increased in a concentration-dependent manner, but with FD4 permeating the barrier to a higher degree than FD10, thus indicating size-selectivity in this molecular size range (Figures 3C–E). These permeation profiles revealed a non-linear relationship between accumulated mass and time with a decrease in flux over time. In contrast, the permeation profiles of FD4 and FD10 during epithelial exposure to EGTA showed steady-state permeation kinetics, except for the 7 mM concentration, for which the permeation rate tended to increase at the later time points and for which the variation in flux also increased (Figures 3F, G). Also, in contrast to that of penetramax, the effect of EGTA did not demonstrate clear size-selectivity, as the calculated P_app_ values were similar for FD4 and FD10 applied together with EGTA (Figure 3H).

**FIGURE 4:**
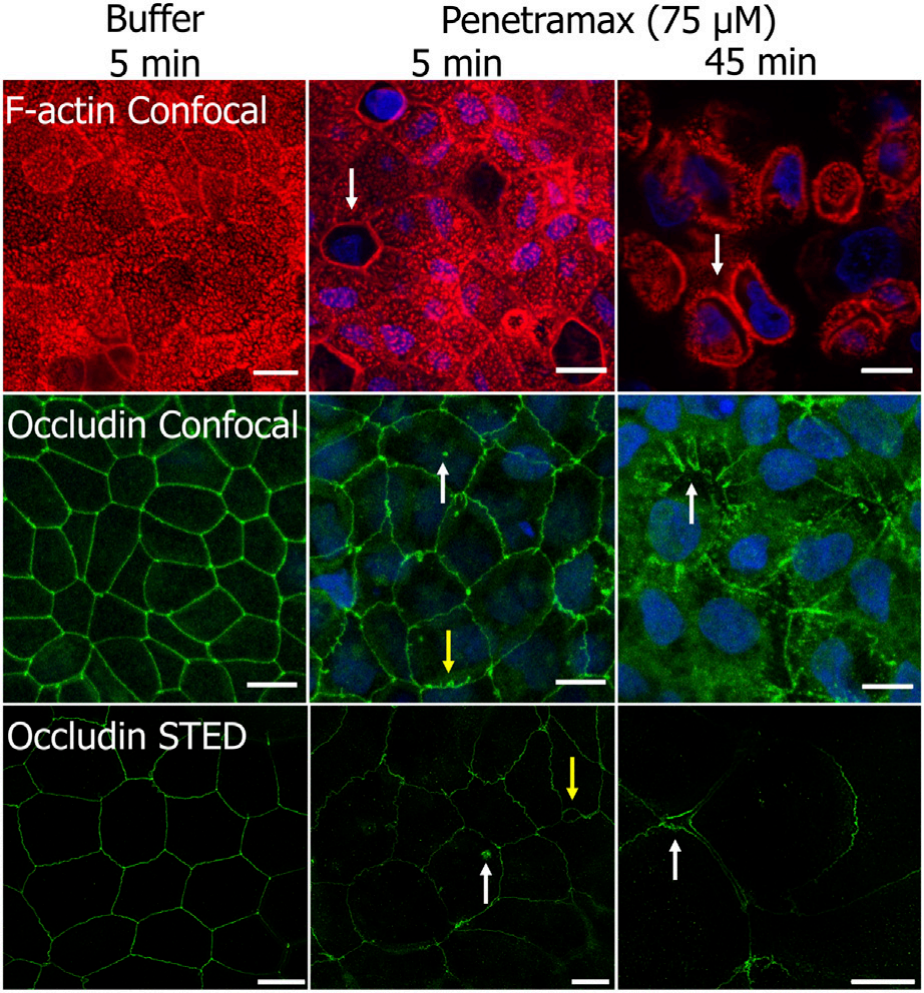
Penetramax at high concentration (75 µM) contracts the F-actin cytoskeleton and widens bicellular TJs (bTJs) and tricellular TJs (tTJs) after 45 min exposure (top row). Internalization of occludin (mid and bottom rows, white arrow) and widening of bTJs (yellow arrows) after 5 min, followed by widening of tTJs after 45 min (white arrows) occurs after exposure to 75 µM penetramax. Representative confocal and STED images of Caco-2 cell monolayers stained for F-actin (confocal) and occludin (confocal and STED) after exposure to buffer for 5 min, 75 µM penetramax for 5 min, and for 45 min (F-actin in red, occludin in green for confocal and STED, nuclei in blue). Confocal. N = 2, *n* = 1 (scale bar 10 µm), STED; N = 1, *n* = 1 (scale bar 5 µm).

To gain further insight into the fast-acting mechanism of penetramax, F-actin and occludin were stained in Caco-2 cell monolayers exposed to 75 µM penetramax for just 5 and 45 min (Figure 4). During that period, penetramax exerted its most pronounced and immediate effect on the TEER (Figure 3A). Upon exposure to penetramax for 5 min (Figure 4), microvilli appearance changed in a small population of cells, and cytoskeletal contraction was observed after 45 min. After exposure to penetramax for 5 min, the characteristic punctate localization of occludin in tricellular TJs (tTJs), the meeting point of three epithelial cells, changed to a bicellular TJ (bTJ) pattern (Figure 4, arrow) and after 45 min, occludin internalized into the cytosol. STED microscopy analysis revealed that bTJs widened after only 5 min exposure to penetramax, along with internalization of occludin, followed by widening of also the tTJs after 45 min.

Taken together, these data show that penetramax elicits a selective, rapid, and transient widening of the bicellular and tricellular space, while the effect of EGTA is slower-acting and without size selectivity, but rapidly reversible.

### 3.4 Penetramax and EGTA exposure modulate epithelial TJ proteins differently

**FIGURE 5:**
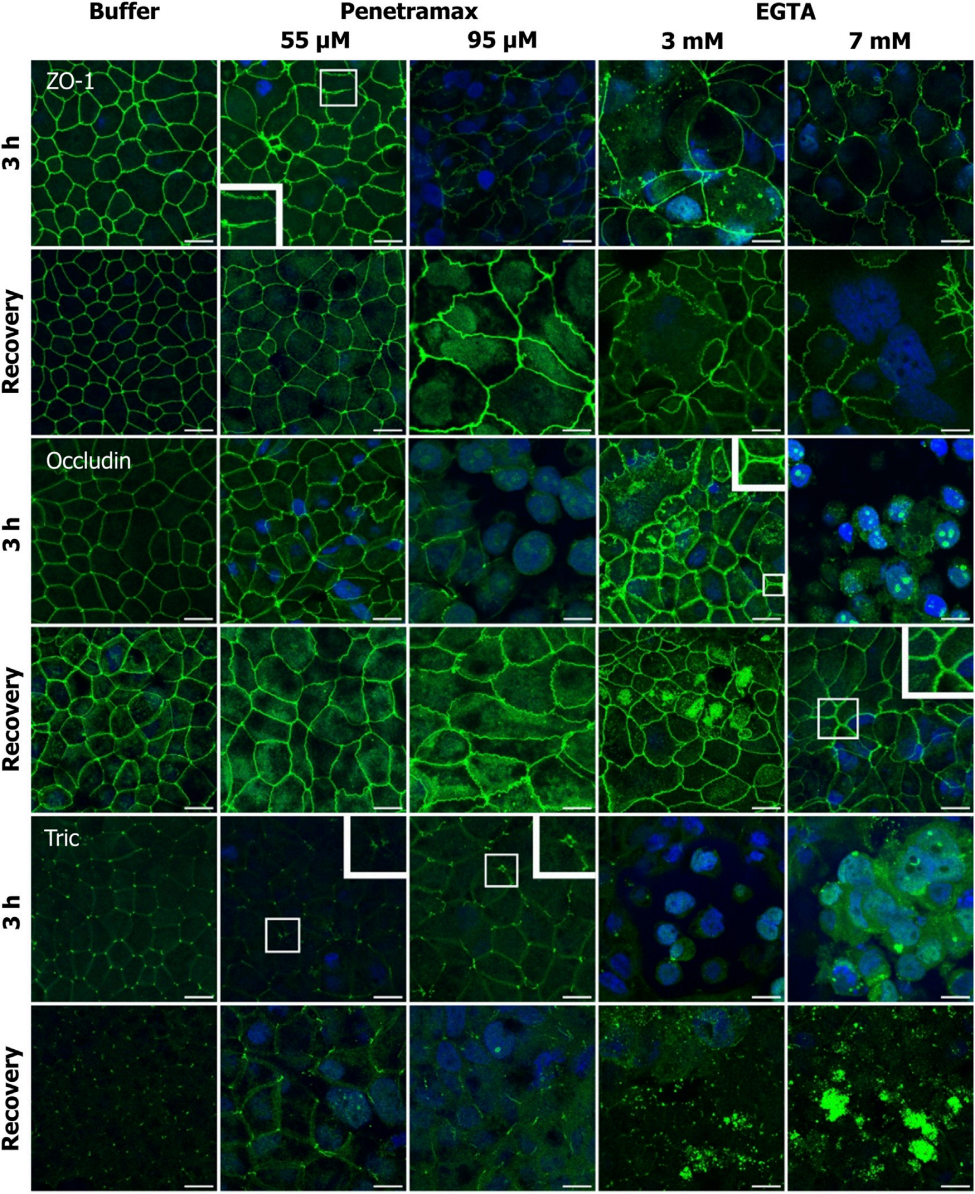
Penetramax alters the TJ morphology, resulting in widening of the bTJs and tTJs, while EGTA induces translocation of TJs intracellularly. Upon recovery, both bTJs and tTJs are closed after penetramax exposure, whereas TJs are not localized on the apical functional network after EGTA exposure. Representative immunofluorescence microscopy images as maximum intensity projections for ZO-1, occludin, and tric after exposure for 3 h (top rows) and after recovery (bottom rows) for penetramax (left columns) and EGTA (right columns). bTJ and tTJ in green, nuclei in blue. N = 3, *n* = 1 for penetramax N = 2–3, *n* = 1 for EGTA. Scale bar = 10 µm.

**FIGURE 6:**
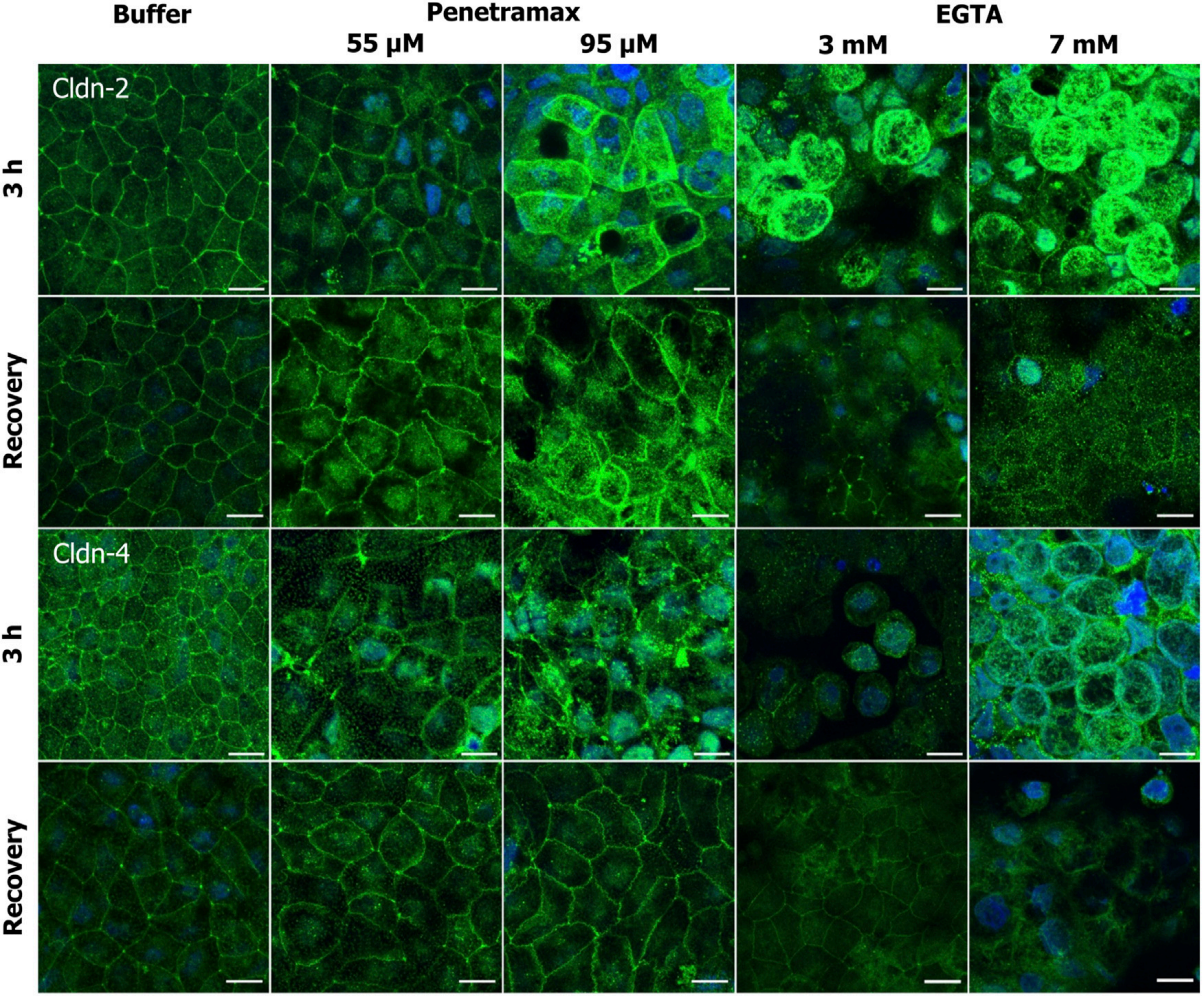
Penetramax exposure induces reorganization of the TJ network by an increase in cldn-2 and decrease in cldn-4 presence in the apical membrane whereas EGTA leads to cytosolic localization of cldn-2 and -4. Representative immunofluorescence microscopy images as maximum intensity projections for cldn-2 and -4 after exposure of 3 h (top rows) and after recovery (bottom rows) for penetramax (left columns) and EGTA (right columns). Cldns in green, nuclei in blue. N = 2–3, *n* = 1. Scale bar = 10 µm.

To further understand the molecular events originating from exposure of Caco-2 cell monolayers to penetramax and EGTA, the localization of ZO-1, occludin, tric, and selected claudins (cldns) were investigated (Figure 5; 6; Supplementary Figures S3–S5).

Immunostaining of ZO-1 revealed alterations in the cell-cell contact morphology upon exposure to penetramax concentrations higher than 25 µM (Figure 5, row 1; Supplementary Figure S3). Most notably, a widening in the paracellular space of the bTJs and a ruffled TJ morphology were observed after exposure to penetramax concentrations of 55 µM or higher. Likewise, the occludin immunostaining (Figure 5, row 3) revealed a ruffled TJ morphology after exposure to 55 µM penetramax (magnified). At higher concentrations, occludin was observed in the nucleus and was reduced in the TJ complex (Figure 5, row 3; Supplementary Figure S3). The effects of penetramax on tTJs were assessed based on the localization of tric (Figure 5, row 5). Exposure to concentrations higher than 25 µM penetramax showed widening of tTJs (Figure 5, row 1, magnified for 55 µM).

We next addressed the cldn localization (Figure 6). Exposure to penetramax for 3 h induced redistribution of cldn-2, -4, and -7, but not of cldn-1 (Figure 6; Supplementary Figures S4, S5). Cldn-2 localized both intracellularly and in the TJ complex, especially after exposure to 95 µM penetramax (Figure 6, row 1). Cldn-4 localized in tTJs to a greater extent upon exposure to 55 µM penetramax when compared to the buffer exposed cell monolayers, whereas at 95 µM penetramax, cldn-4 tended to internalize into the cytosol (Figure 6, row 3).

Exposure to EGTA for 3 h at concentrations exceeding 1 mM altered the morphology of Caco-2 cell monolayers (Figures 5, 6; Supplementary Figures S3–S5). A ruffled TJ morphology of the cell-cell contacts was observed at 7 mM EGTA when stained against ZO-1 (Figure 5; Supplementary Figure S3). To that extent, intracellular localization of ZO-1 and occludin was also observed at 3 mM and 7 mM EGTA, respectively (Figure 5, rows 1 and 3). Further, an apparent widening of the paracellular space (Figure 5, magnified) was observed at 3 mM EGTA when stained against occludin. Lastly, tric and cldn-1, -2, -4 and -7 immunostainings revealed similarly altered phenotypes. A more dramatic loss of the TJ from the plasma membrane was observed at 7 mM EGTA (Figures 5, 6; Supplementary Figures S3–S5).

Upon a recovery phase, the modulation of the paracellular space induced by penetramax was restored. Cldn-4 and occludin relocalized to the TJ complex, yet the ruffled TJ morphology remained as seen in images of ZO-1 and occludin immunostainings. Similarly, cldn-1 and -2 immunostainings revealed signs of restoration yet to a lesser extent than the rest of the investigated TJ proteins for the highest penetramax concentration (Figure 6; Supplementary Figure S4). Lastly, tric was found intracellularly in addition to its presence in tTJs. In contrast, after the recovery phase following EGTA exposure, the modulation of the paracellular space was not similarly restored. The TJs network was absent in some cells as shown when stained against ZO-1 while, cldn-1, -2, -4, and -7 along with tric were found intracellularly and still lost from the plasma membrane (Figures 5, 6; Supplementary Figures S3–S5). Lastly, the paracellular space remained open as seen in cells stained against occludin (Figure 5, row 4, 7 mM EGTA).

**FIGURE 7:**
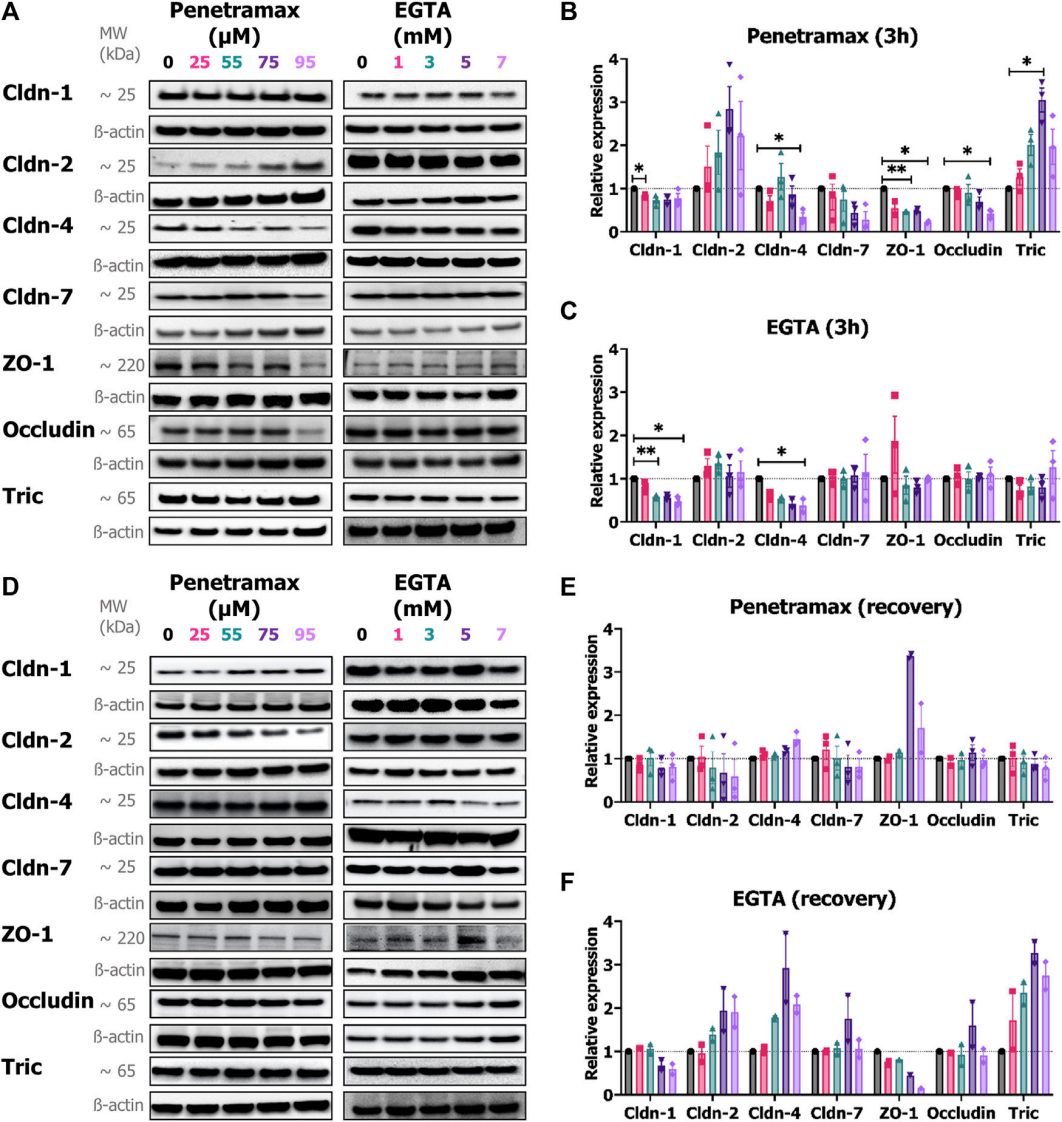
Penetramax alters the expression of TJs upon 3 h of exposure whereas EGTA effects are observed upon recovery. Representative immunoblots of the expression of cldn-1, -2, -4, -7, ZO-1, occludin, and tric after **(A)** 3 h of exposure followed by the relative expression of **(B)** penetramax and **(C)** EGTA after 3 h of exposure and after **(D)** recovery followed by the relative expression of **(E)** penetramax and **(F)** EGTA. N = 2–3, *n* = 6–12. Results are shown as mean ± SD. For quantification, densitometric intensities were normalized to ß-actin loading control displayed for each lane. *) *p* < 0.05, **) *p* < 0.01.

To complement the immunostaining study on localization of the bTJ and tTJ in the cell monolayers, their expression levels were determined. Immunoblot analysis showed that exposure to penetramax was accompanied by a significant concentration-dependent reduction in the overall expression of ZO-1, occludin, cldn-1, and the barrier-forming cldn-4, and -7 (Figures 7A, B). The highest effects were observed after 95 µM penetramax exposure with 77%, 58%, 25%, 66% and 71% reduction for ZO-1, occludin, cldn-1, -4 and -7, respectively. In contrast, expressions of tric and the pore-forming cldn-2 were increased by a factor of 2.0, 3.1, 2.0 and 1.8, 2.8, and 2.2 after exposure to 55, 75, and 95 µM penetramax, respectively (Figures 7A, B). Upon recovery, the expression of the investigated TJ proteins overall returned to baseline values (Figures 7D, E), except for ZO-1 that increased 3.4- and 1.7-fold in cells exposed to 75 and 95 µM penetramax, respectively along with signs of cldn-2 downregulation.

In contrast to penetramax, EGTA exposure altered only the expression of cldn-1 and cldn-4 after 3 h exposure (Figures 7A, C). Interestingly, after the recovery phase, the expression of tric, cldn-2, and cldn-4 increased to up to 3-fold higher levels than the buffer exposed cells, whereas cldn-1 expression remained decreased in samples exposed to 5 and 7 mM EGTA along with a concentration-dependent decrease in expression of ZO-1 (Figures 7D, F).

Taken together, these results illustrate how widening of the paracellular space induced by penetramax exposure is accompanied by immediate yet transient changes in the localization and expression of the tested TJ proteins. In contrast, EGTA exposure impaired the organization of the TJs after 3 h of exposure and this was not restored upon recovery.

## 4 Discussion

Improved absorption of biopharmaceuticals across the tight epithelium of the gastrointestinal tract is pursued, among other strategies, by modulating TJ proteins for increasing paracellular permeation (Neuhaus et al., 2018; Bocsik et al., 2019). In this study, we compared head-to-head the membrane-interacting peptide, penetramax, with the small-molecule and divalent cation chelator, EGTA, as PEs for oral peptide delivery. Specifically, epithelial integrity kinetics in real time, TJ protein expression and localization, and the overall ultrastructure of epithelial cell monolayers after excipient exposure and upon recovery were investigated along with permeability studies to understand the underlying mechanisms related to efficacy and safety of these excipients.

### 4.1 Penetramax exerts specific effects on expression and localization of different TJ proteins

We have previously hypothesized that penetramax acts on TJ proteins due to significant decreases in TEER and corresponding increases in paracellular marker permeation across epithelium exposed to penetramax (Diedrichsen et al., 2021). Our results here demonstrate that penetramax exerts an immediate, transient, and reversible widening of the paracellular space between the enterocytes. This is associated with contraction of the actin cytoskeleton, regulation of specific TJ protein expressions, and changes in their localization. As a result of penetramax exposure, the expression of tric and cldn-2, a classified pore-forming cldn, were upregulated and localized in the cell membrane and cytoplasm, whereas the expression of cldn-4 and -7, classified as cation and anion barrier-forming cldns (Weber and Turner, 2017), respectively, along with the expression of ZO-1 and occludin were downregulated. The downregulated TJ proteins were generally removed from the plasma membrane, suggesting a remodeling of the function of the TJ protein network, observed both in the bTJ and tTJ (Figures 2, 4, 6). While Krug et al. (Krug et al., 2009; Krug, 2017) showed that downregulation of tric lead to increased macromolecule permeation through tTJs, penetramax exposure increased macromolecule permeation along with upregulating tric expression, demonstrating that different modulations of this tTJ protein can result in increased membrane permeability. Watson et al. (Watson et al., 2001) and Van Itallie et al. (Van Itallie and Anderson, 2006) showed that upregulation of cldn-2 expression correlated with an increase in the diameter of the size- and ion-selective pore pathway (Weber and Turner, 2017), while overexpression of cldn-1, -4, and -7 decrease the diameter and subsequently, the overall paracellular permeation due to their proposed sealing properties (Le et al., 2021). On the contrary, the leak pathway allows the passage of macromolecules due to the contraction of actin cytoskeleton to a higher degree than the pore pathway (Monaco et al., 2021). Here, the penetramax-mediated modulation of the actin cytoskeleton and TJ proteins, resulting in size-selective permeation of the paracellular markers of FD4 and FD10 (Figure 3), as the P_app_ values for FD10 at all tested penetramax concentrations were lower than those for FD4. Previous work display that the enhanced permeation of FD4 by penetramax to a reasonable extent resembles the effect on insulin permeation, although the latter is slightly lower (Diedrichsen et al., 2021; Diedrichsen et al., 2023), likely due to differences in molecular weight, structure, and net charge. The observed permeation enhancement can be attributed to penetramax-induced TJ remodeling towards the leak pathway since cldn-2 is upregulated while cldn-4 and -7 is downregulated. The transient effect of penetramax during exposure is likely due to its peptidic nature and susceptibility to degradation. In contrast to penetramax, the amphipathic cell-penetrating peptide PN159, which was proposed to be a TJ-modulating peptide by Bocsik et al*.* (Bocsik et al., 2019), also resulted in an immediate reduction in TEER, but its effect was not transient during exposure as for penetramax, and TEER reversed only after medium exchange (Bocsik et al., 2019; Saaber and Reichl, 2019). In addition, PN159 exposure did not result in a size-selective passage of paracellular markers as similar permeation rates for FD4, FD10, and FD20 were determined. This was interpreted as a mechanism involving transcellular perturbation by Maher et al. (Maher et al., 2019). Our results with penetramax show increased TEER upon recovery along with morphological changes in terms of fewer, shorter, and flatter cells in the monolayer (Figures 5, 6; Supplementary Figure S1). However, the expression of the TJ proteins was reversible upon recovery. Thus, the membrane-interacting properties of cell-penetrating peptides (Kamei et al., 2013b; Shi et al., 2014) and resulting rapid effects reported here contrast with the required 12-48 h pre-exposure of other peptides described as TJ modulating peptides, such as angubindin-1 and trictide (Cording et al., 2017; Krug et al., 2017).

### 4.2 Non-specific and time-dependent modulation of TJs by EGTA

EGTA is a well-known Ca^2+^ chelator and modulates the cytoskeleton and the TJ protein network either directly via extracellular depletion of Ca^2+^ or by shifting the extra-to intercellular Ca^2+^ balance (Raiman et al., 2003). Further understanding how EGTA affects TJ and cytoskeleton dynamics and thereby acts as a PE is lacking regardless that its analog EDTA is a constituent in the POD™ technology investigated for oral peptide delivery (Zizzari et al., 2021). We showed here that EGTA induced a gradual and concentration-dependent decrease in epithelial barrier properties during exposure, as determined by TEER measurements (Figure 3). Our results suggest that the chelation of divalent cations results in heterogeneous effects across the monolayer, as previously demonstrated by Tria et al. (Tria et al., 2013). This is supported by the observations by Richter et al. (Richter et al., 2016), who suggested epithelial hot spots for macromolecular permeation induced by EGTA. Importantly, we did not deplete the experimental buffer in terms of divalent cations to better resemble *in vivo* conditions, which contrasts with earlier studies that used Ca^2+^ and Mg^2+^ depleted media when investigating EGTA and EDTA as permeation enhancers (Farshori and Kachar, 1999). The buffer contained 1 mM Ca^2+^ and 0.9 mM Mg^2+^ leading to complexation of nearly all Ca^2+^ ions at the 1 mM EGTA concentration due to a 1:1 M binding ratio and preferential Ca^2+^ over Mg^2+^ binding (Caldwell and Cuthbert, 1970). At the higher EGTA concentrations, all divalent cations are bound in an EGTA complex and as such depleted from the buffer. Correspondingly, our data showed less TEER reduction than previously reported (Farshori and Kachar, 1999; Ma et al., 2000; Rothen-Rutishauser et al., 2002; Yu et al., 2013; Saaber and Reichl, 2019). It is interesting, that despite an apparent disruption of the TJs following exposure to EGTA (Figure 2), the TEER rapidly returned to baseline levels after removal of EGTA (Figure 3B). Epithelial reorganization and/or unaffected cells may compensate for this. For all TJ proteins investigated, a subpopulation of cells (Supplementary Figures S3–S5), in which TJ proteins were localized intracellularly, demonstrated that divalent cation depletion resulted in loss of TJ proteins from the plasma membrane. Following exposure to EGTA, only cldn-1 and -4 showed a significant decrease in expression, thus suggesting their importance for Ca^2+^ regulation. While penetramax had a more significant effect on the permeation of FD4 than that of FD10, EGTA did not show such size selectivity (Figures 3F–H), possibly due to its clearly different effect on the paracellular space despite overall similar TEER levels were determined. Regulation of TJ protein expression occurred during the recovery period after EGTA exposure as revealed by the upregulation of cldn-2, -4, and tric. In addition to this, the localization of all the investigated TJ proteins suggests that the reconstitution mechanism within the Caco-2 cell monolayers after EGTA exposure relies on their *de novo* protein synthesis, implying a longer time required for the recovery (re-epithelization) process in comparison to what was observed after exposure to penetramax.

### 4.3 Reversibility of epithelial barrier integrity after excipient exposure

The role of the actin cytoskeleton and the TJ dynamics in intestinal epithelial regeneration after excipient exposure is poorly understood. The present study aimed to elucidate the underlying mechanism(s) involved in epithelial barrier effects and recovery.

Penetramax reduced epithelial tightness resulting in a transient loss of cell polarity (Figure 2; Supplementary Figures S1, S2) and a partial mesenchymal phenotype (Supplementary Figure S2). Previous studies have addressed alterations in cell morphology and apical-basal polarity (Du et al., 2010), remodeling of the TJs (Balda and Matter, 2016), and induction of cell motility (Yan et al., 2010; Jiang et al., 2018; Dongre and Weinberg, 2019) as part of epithelial-to-mesenchymal transition that occurs during epithelial recovery. Such events were observed after exposure to penetramax as part of the re-epithelialization process. This may explain the observation of stress fibers, essential actin-based components for cellular contractility, migration, and adhesion (Gaston et al., 2021; Lehtimäki et al., 2021), after cytoskeletal re-organization (Figure 2A). Interestingly, after the recovery period following EGTA exposure, the barrier was still compromised as TJ proteins were not localized in the apical-lateral TJ network (e.g., ZO-1) and F-actin disassembly was evident (Figures 2, 5). Like what was observed after exposure to penetramax, the cellular volume after EGTA exposure was smaller than that of cells exposed to buffer (Supplementary Figures S1, S2), likely a result of divalent cation depletion and its effect on the cytoskeletal network.

## 5 Conclusion

In the present study, we demonstrate that the modulation of dynamic properties of the intestinal epithelial barrier is dependent on the excipient type. Penetramax exposure resulted in a fast, transient, and size-selective widening of the paracellular space that corresponded to a transient TJ remodeling. In contrast, EGTA resulted in a gradual and time-dependent effect that was not reflected in TJ protein expression levels and delayed the TJ re-localization to the plasma membrane for sealing the paracellular space. However, it was clear that independently of the excipient type, the TJ modulation events are linked to a transient decrease in cell polarity. Overall, understanding TJ modulation and cytoskeleton dynamics in well-differentiated epithelia may lead to better understanding of parameters relevant for selection of excipients, such as PEs that are desired to rapidly and in a controlled, specific, and reversible manner exert the desired effect leading to selective improved absorption of the biopharmaceutical in question. From this perspective, and based on our findings, penetramax seems to be superior to EGTA. More detailed understanding of cellular responses to PEs will guide the selection of excipients suitable for use in oral peptide delivery.

## Data Availability

The original contributions presented in the study are included in the article/Supplementary Material, further inquiries can be directed to the corresponding author.

## Abbreviations

bTJ, bicellular TJ; Caco-2, colorectal adenocarcinoma cell line; cldn, claudin; CLSM, confocal laser scanning microscopy; EDTA, ethylenediaminetetraacetic acid; EGTA, ethylene glycol tetraacetic acid; F-actin, filamentous actin; FD, stimulation emission depletion microscopy; FITC-dextran; MCFA, medium chain fatty acid; PE, permeation enhancer; STED, stimulation emission depletion microscopy; TEER, transepithelial electrical resistance; TEM, transmission electron microscopy; TJ, tight junction; tric, tricellulin; tTJ, tricellular TJ; ZO, zonula occludens.
