# Supplementary material for: Epithelium dynamics differ in time and space when exposed to the permeation enhancers penetramax and EGTA. A head-to-head mechanistic comparison
Source: Front Drug Deliv. 2023 Aug 24;3:1221628. doi: 10.3389/fddev.2023.1221628 (PMC12363289; doi:10.3389/fddev.2023.1221628)
Supplement: Supplementary file 1 [file Table1.DOCX]

Supplementary Material

Epithelium dynamics differ in time and space when exposed to the permeation enhancers penetramax and EGTA. A head-to-head mechanistic comparison.

**Panou D.A.^1^, Pedersen S. F.^2^, Kristensen M.^3^, Nielsen H.M.^1 *^**

^1^Center for Biopharmaceuticals and Biobarriers in Drug Delivery (BioDelivery), Department of Pharmacy, Faculty of Health and Medical Sciences, University of Copenhagen, Universitetsparken 2, DK-2100 Copenhagen, Denmark

^2^Section for Cell Biology and Physiology, Department of Biology, Faculty of Science, University of Copenhagen, Universitetsparken 13, DK-2100 Copenhagen, Denmark

^3^CNS Drug Delivery and Barrier Modelling, Department of Pharmacy, Faculty of Health and Medical Sciences, University of Copenhagen, Universitetsparken 2, 2100 Copenhagen, Denmark

*Corresponding author

Hanne Mørck Nielsen

Department of Pharmacy, Faculty of Health and Medical Sciences, University of Copenhagen, Universitetsparken 2, DK-2100 Copenhagen, Denmark

E-mail: [hanne.morck@sund.ku.dk](mailto:hanne.morck@sund.ku.dk)


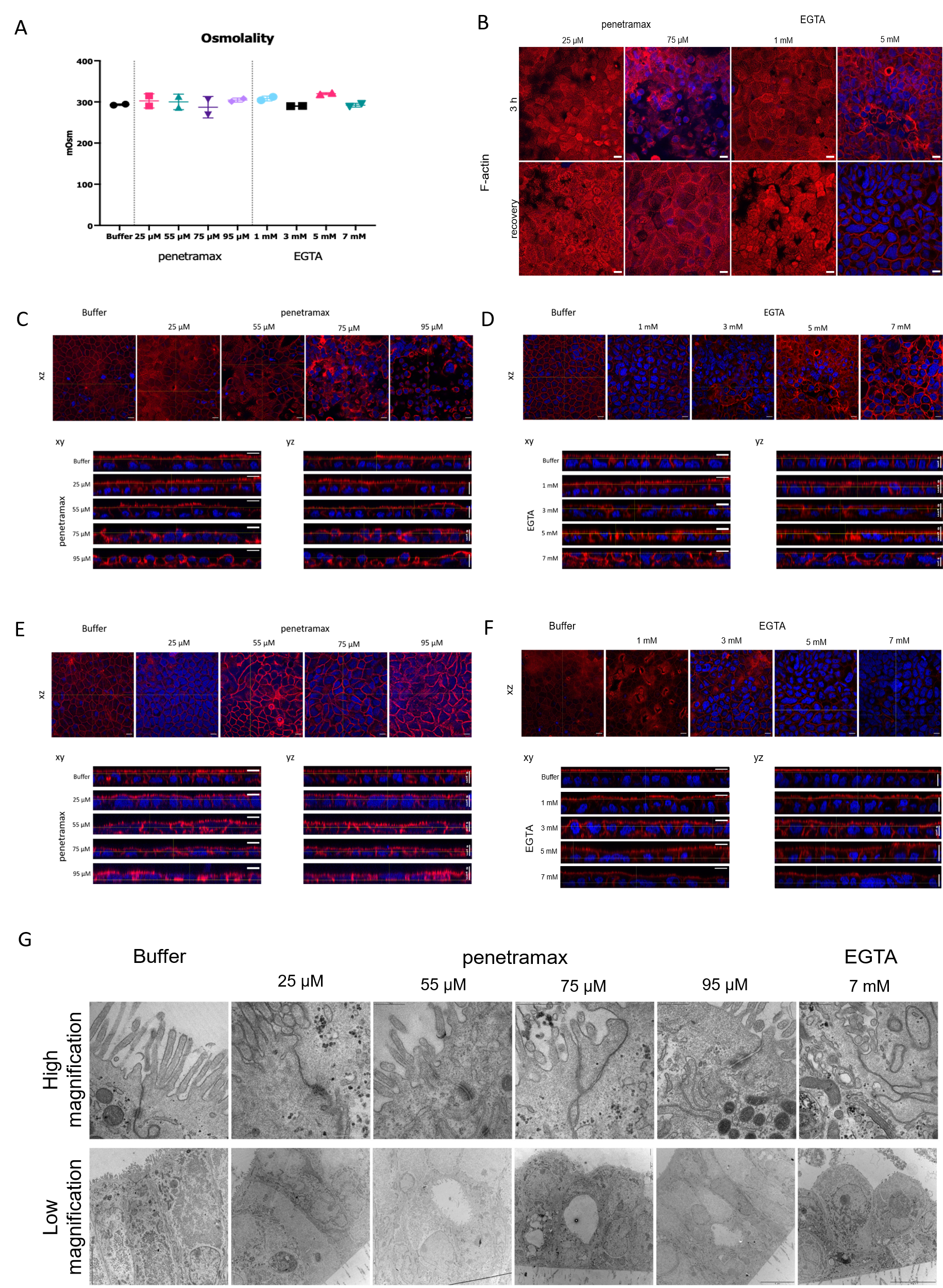


**Figure S1. A) Osmolality measurements for the tested concentrations of the two excipients. The test concentrations of penetramax and EGTA are approximately at 300 mOsm, resulting in iso-osmolar samples. B) Penetramax and EGTA induce the contraction of F-actin resulting in a rounded phenotype. Representative confocal images of rhodamine phalloidin for visualization of F-actin as maximum projections (3 planes) after penetramax (25 µM and 75 µM) and EGTA (1 mM and 5 mM) exposure for 3 h (top) or upon recovery (bottom). C-F) Complementary orthogonal views presenting the xy, xz, and yz dimensions of Caco-2 cell monolayers that have been exposed to penetramax C) after 3 h and E) upon recovery and EGTA D) after 3 h and F) upon recovery. Scale bar 10 µm otherwise stated. N=2-3.**

*
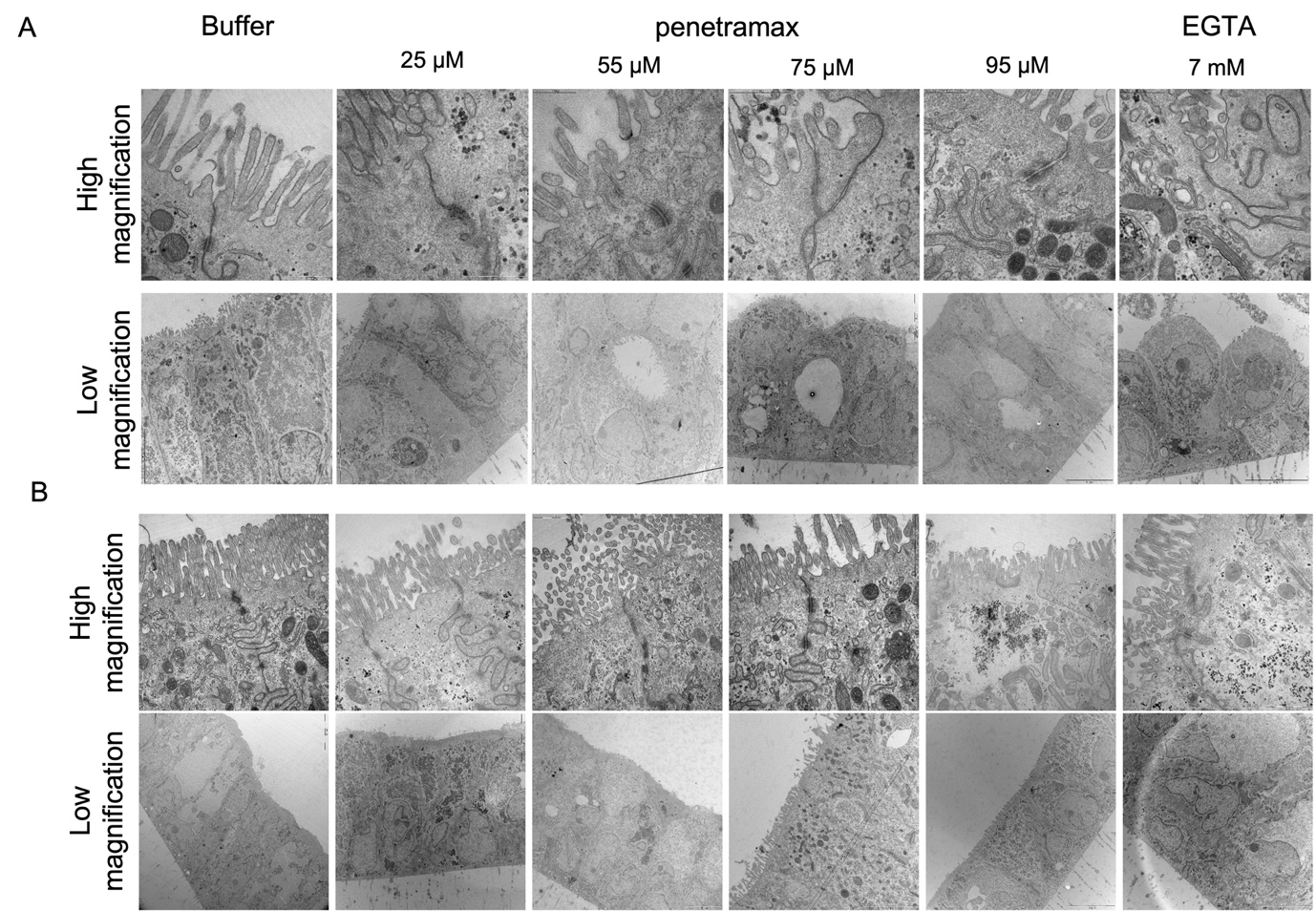
*

Figure S2. The effect of all the tested concentrations of penetramax illustrated in high magnification depicting the widening of the paracellular space while TJs are present in contrast to the EGTA induced opening of the paracellular space. Low magnification shows the effect of penetramax on inducing cytoskeletal re-arrangement linked with cell migration and alteration of the Caco-2 monolayer. In contrast, EGTA dissociates cells from the monolayer and TJs are seen in the basal compartment. Upon recovery, TJs are present in both tested excipients however in low magnification the heigh of the monolayer seen altered in 95 µM penetramax. In addition, the heterogeneity of EGTA can be seen in low magnification as Caco-2 cells are in multilayer without TJs. A and B) Representative transmission electron microscopy images in high and low magnification. Scale bar for high magnification 1 µm and low magnification 10 µm apart from 75 µM (scale bar 5 µm).

**Table S1. Overview of replicates in permeation study. N corresponds to passage number and n corresponds to individual filter inserts per passage. The exact time point where samples were excluded (leading to N=2 for that time point only) are shown.**

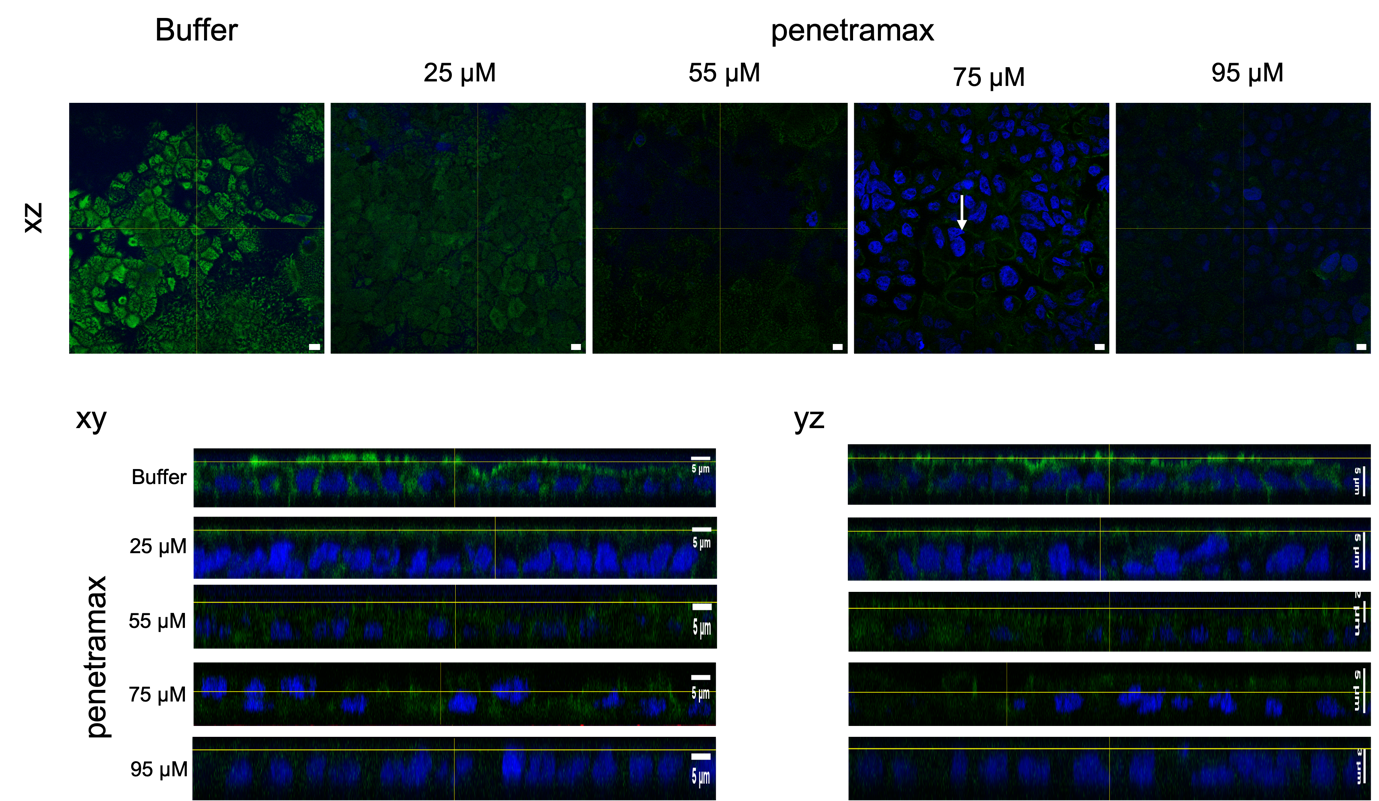


Figure S3. The contraction of F-actin cytoskeleton (green) results in the widening for the transport of macromolecules of approximately 10 kDa (arrow), indicating a paracellular mechanism of action additionally to the proposed internalization mechanism (xy and yz). Orthogonal views of Caco-2 monolayers after 3 h exposure to buffer, 25 µM, 55 µM, 75 µM, and 95 µM penetramax with FITC-dextran 10 for transport studies. Scale bar 5 µm if not otherwise stated.


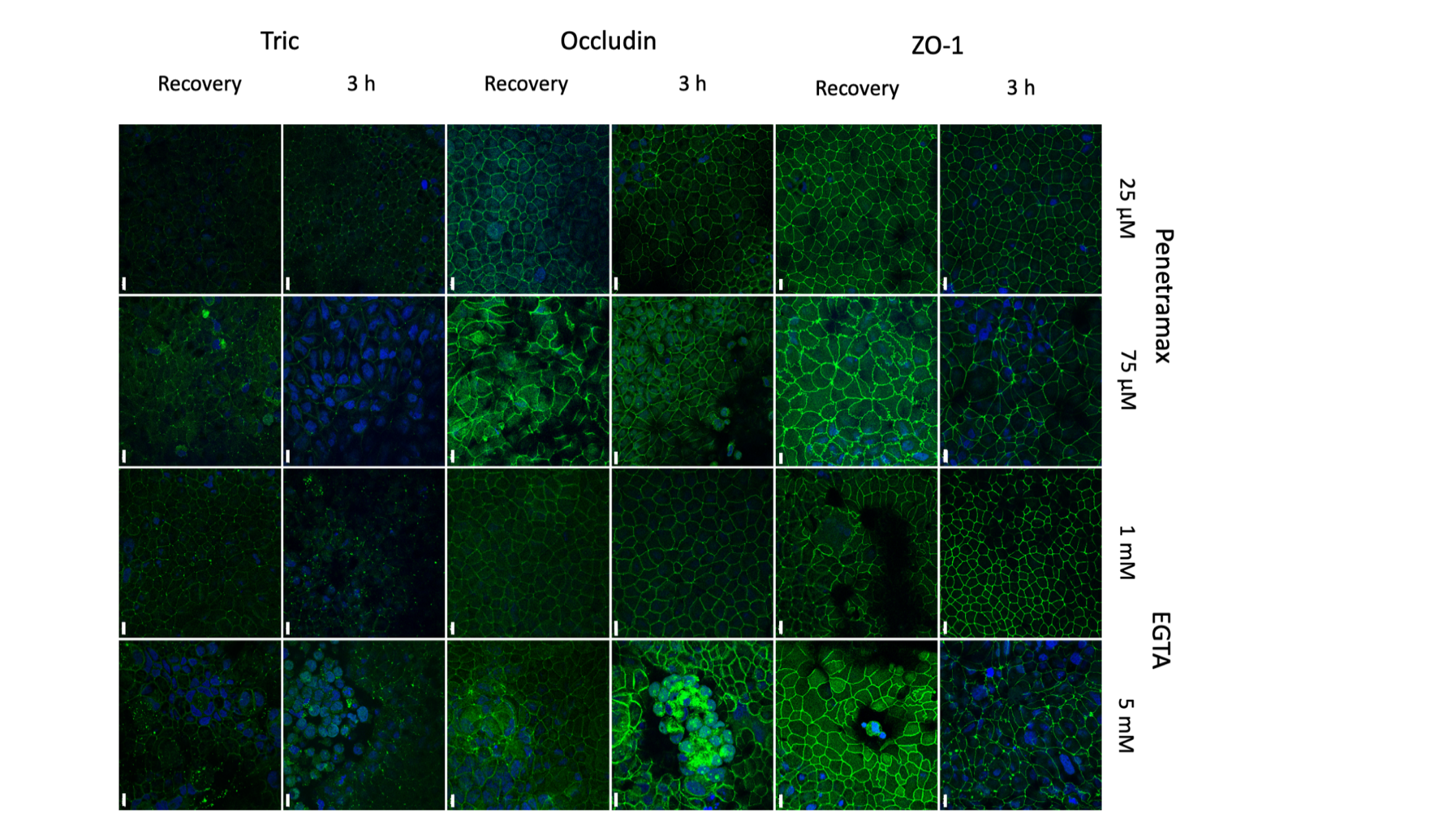


Figure S4. The effect of penetramax and EGTA on TJ localization for the low and intermediate high concentrations 25 µM, 75 µM and 1 mM, 5 mM, respectively. Representative immunofluorescence microscopy images as maximum intensity projections for ZO-1, occludin, and tric after exposure of 3 h (top rows) and after recovery (bottom rows) for penetramax (left columns) and EGTA (right columns). N=3, n=1 for penetramax N=2-3, n=1 for EGTA. Scale bar= 10 µm.


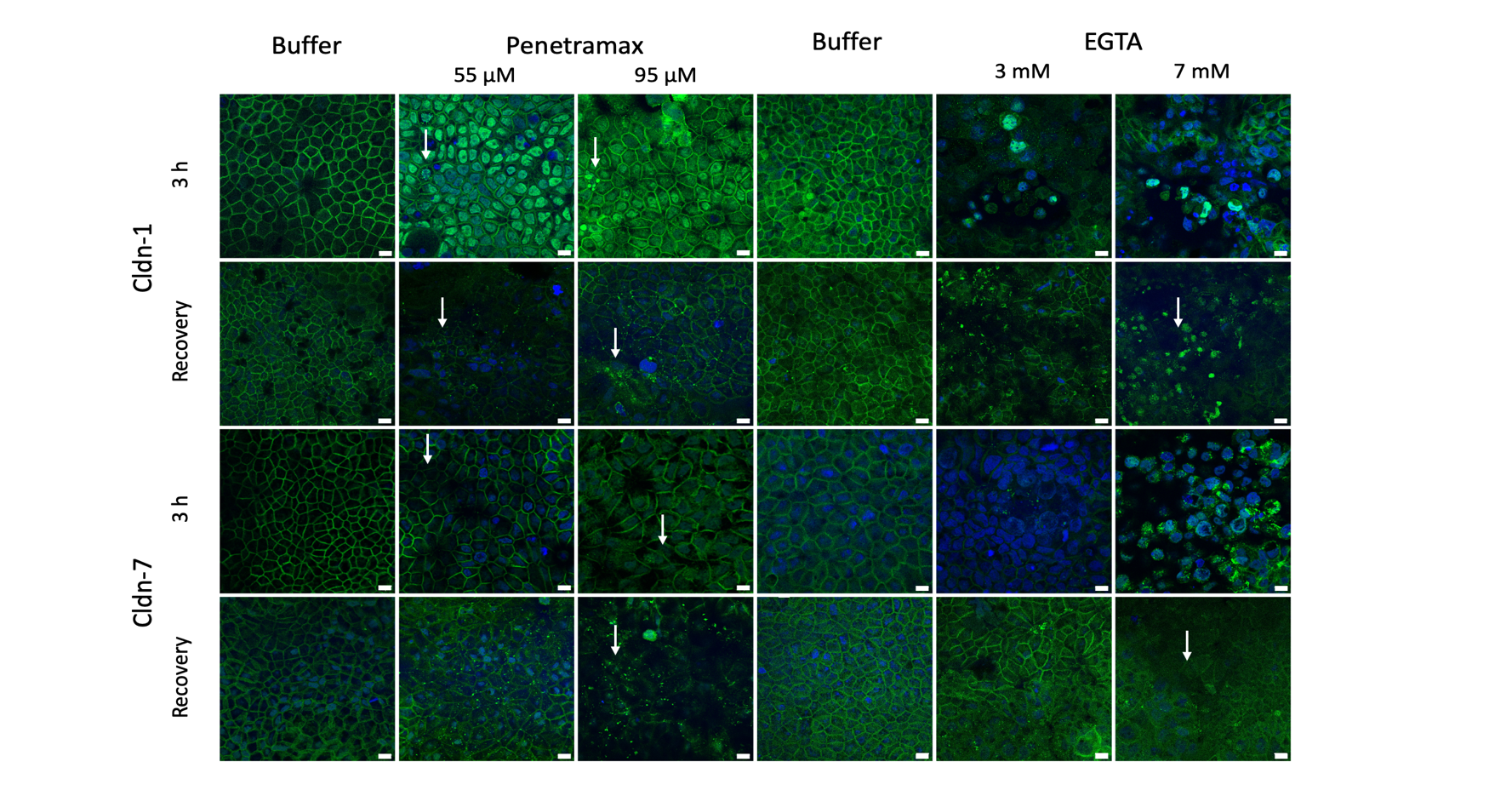


Figure S5. Penetramax induces the reorganization of TJ network by depleting cldn-7 from the plasma membrane and the continuous expression of cldn-1 (arrows) whereas, EGTA translocates TJs from the plasma membrane intracellularly. Upon recovery, cldn-1 and -7 are primarily found intracellularly for the high concentration of penetramax and EGTA. Representative immunofluorescence microscopy images as maximum intensity projections for cldn-1 and -7 after exposure of 3 h (top rows) and after recovery (bottom rows) for penetramax (left columns) and EGTA (right columns). N=2-3, n=1. Scale bar= 10 µm.


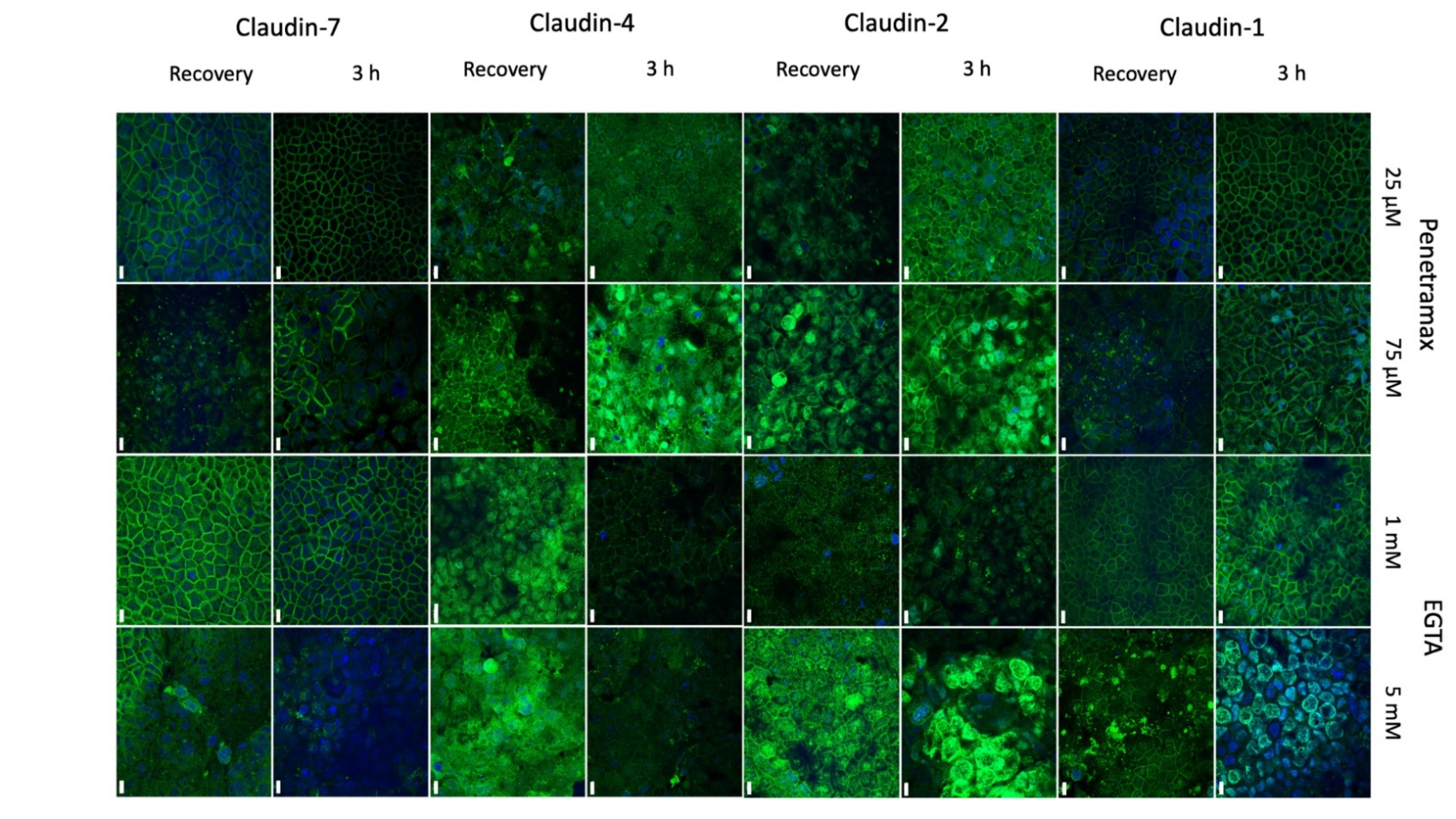


Figure S6. The effect of penetramax and EGTA on TJ localization for the low and intermediate high concentrations 25 µM, 75 µM and 1 mM, 5 mM, respectively. Representative immunofluorescence microscopy images as maximum intensity projections for cldn-1, -2, -4, and -7 after exposure of 3 h (top rows) and after recovery (bottom rows) for penetramax (left columns) and EGTA (right columns). N=3, n=1 for penetramax N=2-3, n=1 for EGTA. Scale bar= 10 µm.

**
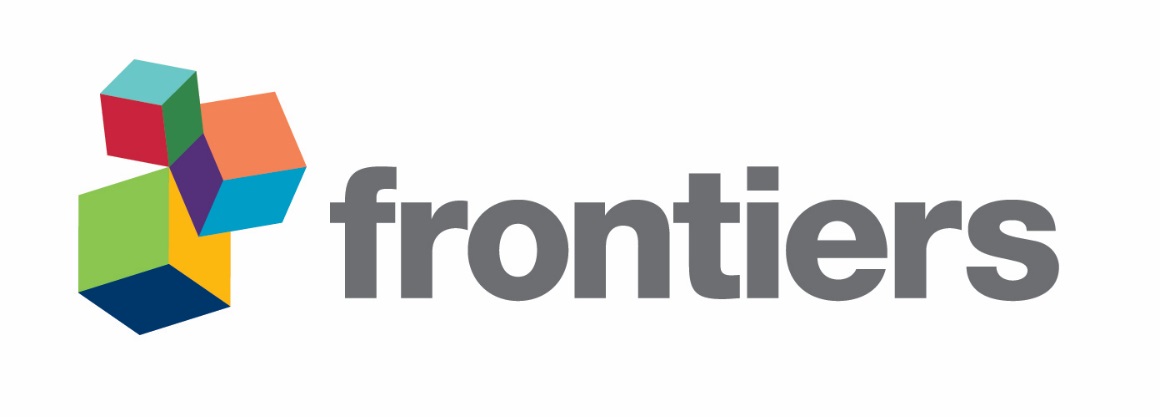
**
