# Supplementary figures and images for: Epithelium dynamics differ in time and space when exposed to the permeation enhancers penetramax and EGTA. A head-to-head mechanistic comparison
Source: Front Drug Deliv. 2023 Aug 24;3:1221628. doi: 10.3389/fddev.2023.1221628 (PMC12363289; doi:10.3389/fddev.2023.1221628)

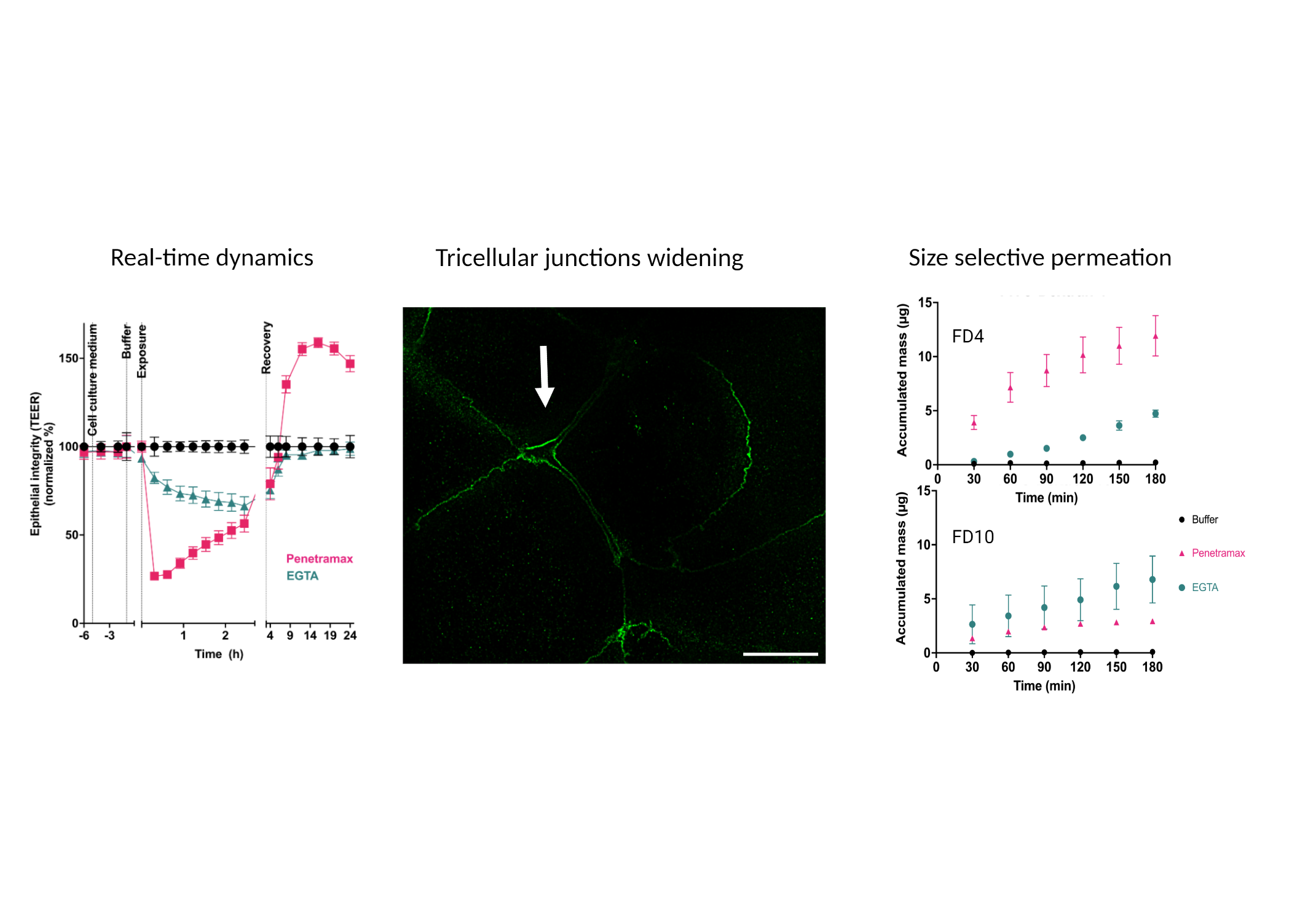

Supplement: Supplementary file 2 [file Image1.PNG]
